# HMGA2 Antisense Long Non-coding RNAs as New Players in the Regulation of HMGA2 Expression and Pancreatic Cancer Promotion

**DOI:** 10.3389/fonc.2019.01526

**Published:** 2020-01-17

**Authors:** Gloria Ros, Silvia Pegoraro, Paolo De Angelis, Riccardo Sgarra, Silvia Zucchelli, Stefano Gustincich, Guidalberto Manfioletti

**Affiliations:** ^1^Department of Life Sciences, University of Trieste, Trieste, Italy; ^2^Department of Health Sciences, Center for Autoimmune and Allergic Diseases, Interdisciplinary Research Center of Autoimmune Diseases, University of Piemonte Orientale, Novara, Italy; ^3^Central RNA Laboratory, Istituto Italiano di Tecnologia, Genoa, Italy

**Keywords:** natural antisense non-coding RNAs, HMGA, cancer, gene expression regulation, FANTOM5, HMGA2-AS1

## Abstract

**Background:** Natural antisense long non-coding RNAs (lncRNAs) are regulatory RNAs transcribed from the opposite strand of either protein coding or non-coding genes, able to modulate their own sense gene expression. Hence, their dysregulation can lead to pathologic processes. Cancer is a complex class of diseases determined by the aberrant expression of a variety of factors, among them, the oncofetal chromatin architectural proteins High Mobility Group A (HMGA) modulate several cancer hallmarks. Thus, we decided to investigate the presence of natural antisense lncRNAs in *HMGA1* and *HMGA2 loci*, and their possible involvement in gene expression regulation.

**Methods:** We used FANTOM5 data resources, FANTOM-CAT genome browser and Zenbu visualization tool, which employ 1,829 human CAGE and RNA-sequencing libraries, to determine expression, ontology enrichment, and dynamic regulation of natural antisense lncRNAs in *HMGA1* and *HMGA2 loci*. We then performed qRT-PCR in different cancer cell lines to validate the existence of HMGA2-AS1 transcripts. We depleted HMGA2-AS1 transcripts with siRNAs and investigated HMGA2 expression by qRT-PCR and western blot analyses. Moreover, we evaluated cell viability and migration by MTS and transwell assays, and EMT markers by qRT-PCR and immunofluorescence. Furthermore, we used bioinformatics approaches to evaluate HMGA2 and HMGA2-AS1 correlation and overall survival in tumor patients.

**Results:** We found the presence of a promoter-associated lncRNA (*CATG00000088127.1*) in the *HMGA1* gene and three antisense genes (*RPSAP52, HMGA2-AS1*, and *RP11-366L20.3*) in the *HMGA2* gene. We studied the uncharacterized HMGA2-AS1 transcripts, validating their existence in cancer cell lines and observing a positive correlation between HMGA2 and HMGA2-AS1 expression in a cancer-derived patient dataset. We showed that HMGA2-AS1 transcripts positively modulate HMGA2 expression and migration properties of PANC1 cells through HMGA2. In addition, Kaplan-Meier analysis showed that high level of HMGA2-AS1 is a negative prognostic factor in pancreatic cancer patients.

**Conclusions:** Our results describe novel antisense lncRNAs associated with *HMGA1* and *HMGA2* genes. In particular, we demonstrate that HMGA2-AS1 is involved in the regulation of its own sense gene expression, mediating tumorigenesis. Thus, we highlight a new layer of complexity in the regulation of HMGA2 expression, providing new potential targets for cancer therapy.

## Introduction

The advent of next-generation high throughput sequencing highlighted a new regulation layer in which RNA is a fundamental player. In fact, despite proteins were considered as final effectors in all cell regulation aspects, RNA molecules and especially non-coding RNAs have emerged as crucial and active players in cell orchestration, in particular in gene expression regulation (1–3). Non-coding RNAs are usually classified based on their length, with an artificial cutoff of 200 nucleotides (nt), in small non-coding RNAs (sncRNA) shorter than 200 nt and in long non-coding RNAs (lncRNAs) longer than 200 nt (4). LncRNAs represent a heterogeneous family and can be classified according to their position and transcription direction relative to nearby genes. Among them, natural antisense lncRNAs are characterized by being transcribed from the opposite strand of a protein-coding gene (5–8). Natural antisense lncRNAs can stimulate or reduce the gene expression of the sense transcripts at multiple levels, assuming a functional role in physiological and pathological processes (8–11).

The FANTOM5 Consortium has profiled almost 2,000 human samples from cell lines, primary cells, and tissues, using Cap Analysis of Gene Expression (CAGE) coupled to single-molecule sequencing (12), to accurately map Transcription Start Sites (TSS) and gene regulatory elements and to compile a comprehensive promoter-level mammalian expression atlas. Recently, the FANTOM5 Consortium has further expanded expression datasets, profiling thousands of samples with RNA and short RNA sequencing and paired-end CAGE (CAGEscan) protocols, to generate additional atlases of lncRNAs and miRNAs, mapping their promoters, improving annotation and providing cues of their regulatory functions (8, 13). Altogether, data from the FANTOM5 provide an invaluable tool to identify novel antisense lncRNAs with potential regulatory functions and disease association.

HMGA (High Mobility Group A) proteins are chromatin architectural factors involved in modulating the expression of a broad range of genes (14, 15). Despite HMGA proteins are not able to intrinsically trans-activate gene expression, their plasticity in binding DNA and/or transcription factors (16, 17), makes them key elements in a wide variety of biological processes (18). In physiological conditions, HMGA proteins exert their role of architectural transcription factors during embryogenesis, where they are mainly expressed. In adult tissues these proteins are almost undetectable except in cancer cells, where HMGA are over-expressed and crucial for tumor onset and progression (19, 20). In fact, HMGA drive tumor progression through the modulation of several hallmarks of cancer, such as cell proliferation, metastatic processes, drug resistance and stem cell properties (21–30). Human HMGA proteins are encoded by two distinct paralogous genes: *HMGA1*, that extends for 10 kb on chromosome 6 (6p21) and *HMGA2* that is a 160 kb long gene located on chromosome 12 (12q14-15) (20). The expression of these two genes is orchestrated both at transcriptional and post-transcriptional level (28, 31, 32). In addition, very recently, two research groups revealed that ribosomal protein SA pseudogene (RPSAP52) antisense lncRNA at the 5′ of *HMGA2* gene is able to modulate HMGA2 both at transcriptional (33) and post-transcriptional level (34, 35).

Considering the increasing importance of the antisense lncRNAs in the regulation of coding genes and their involvement in cancer progression through the modulation of crucial oncogenes and oncosuppressors and taking advantage of the genome-wide expression datasets of the FANTOM5 Consortium, we decided to evaluate the presence, expression profile and functional potentials of previously unidentified antisense lncRNAs in *HMGA1* and *HMGA2 loci*. We found novel antisense lncRNAs at both genes. In particular, we showed that a natural antisense lncRNA gene in *HMGA2 locus, HMGA2-AS1*, expresses a number of transcript variants involved in the regulation of sense protein-coding *HMGA2* gene. Moreover, we demonstrated that they have a role in tumorigenesis via an HMGA2-dependent mechanism. The findings reported in this paper add a further layer of complexity to the regulation of HMGA2 expression by previously uncharacterized natural antisense lncRNAs.

## Materials and Methods

### Cell Culture

Human breast cancer MDA-MB 231 and MDA-MB 157, pancreatic cancer BX-PC3 and PANC1, colon cancer SW480 and HCT116, thyroid tumor ARO and TPC1, liver cancer HepG2 and Hep3B, and prostate cancer DU145 cell lines were cultured in DMEM (EuroClone: ECB7501L), whereas prostate cancer PC3 were cultured in RPMI (EuroClone: ECB9006L). Both media were supplemented with 10% tetracycline-free FBS (EuroClone: ECS0182L), L-Glutamine 2 mM (EuroClone: ECB3000D), Penicillin 100 U/ml and Streptomycin 100 μg/ml (EuroClone: ECB3001D).

### Cell Transfections and Treatments

For silencing experiments in PANC1 cells, 2.1^*^10^4^ cells/cm^2^ were treated with 12 nM of siRNA and LipofectamineTM RNAiMAX reagent (Invitrogen: 13778075) according to manufacturer instructions, for 24, 48, 72 h, depending to experiment. siCTRL was already used before (26), siHMGA2-AS1-AGI (5′-GGTGATGTATGGCCCATAA-3′) and siHMGA2-AS1-all (5′-GGGCCAACATGACACCAAA-3′) were designed using Primer Designer Tool from Thermo Fisher Scientific.

We used the following plasmids: pcDNA3.1, pEGFP-N1 (Invitrogen), pEGFP-N1-HMGA2, already available in the laboratory (36), pcDNA3.1-A2-AS1_H and pcDNA3.1-A2-AS1_G. To clone A2-AS1_H (FTMT24500018418.1), and A2-AS1_G (ENST00000536648.1) we amplified them, using primer forward 5′-CCCGCAAGCTTATAACTGGATCTTTCCATTACTTGGTAGC-3′ and primer reverse 5′-AAAGGTACCCTGAGATGCAGCTGACATGTACCA-3′, from cDNA retrotranscribed from PANC1 total RNA, then we purified the two PCR products after separation on agarose gel and we cloned them into pcDNA3.1. For A2-AS1_H and A2-AS1_G overexpression, 3.6^*^10^4^ cells/cm^2^ PANC1 cells were transfected with 1.25 μg/ml of pcDNA3.1 as control and pcDNA3.1-A2-AS1_H or pcDNA3.1-A2-AS1_G, using LipofectamineTM 3000 (Invitrogen: L3000008) according to manufacturer instructions, for 30 h.

For rescue functional analysis, 4.0^*^10^4^ cells/cm^2^ PANC1 cells were plated. Then cells were cotransfected, at 24 and 48 h from seeding, with 12 nM siRNA (siCTRL or siHMGA2-AS1-all) and 1.2 μg/ml of plasmid DNA (pEGFP-N1 or pEGFP-N1-HMGA2), using LipofectamineTM 3000 (Invitrogen). Experiments were done 72 h from the first transfection.

All transfections were performed in DMEM 10% tetracycline-free FBS, L-Glutamine 2 mM (EuroClone).

### Immunoblotting

Cells were washed in chilled PBS and lysed using TRIzol® Reagent (Ambion® by Life Technologies: 15596026) or SDS sample buffer [62.5 mM Tris pH 6.8; 2% SDS; 10% glycerol; 50 mM DTT; Na_3_Vo_4_ 1 mM; NaF 5 mM; PIC mammals (Sigma: P8340)]. In the case of TRIzol® Reagent usage, proteins were extracted accordingly to manufacturer instructions. The use of TRIzol® Reagent allowed to extract both RNA and proteins from the same sample. Lysates were separated by SDS-PAGE, prior to transfer to nitrocellulose membranes (GE-healthcare: GEH10600001). Western blot analyses were performed according to standard procedures using the following antibodies: anti-HMGA2 (37) and anti-β-actin (Sigma: A2066).

### Immunostaining

Immunostaining was performed as described previously (30). E-Cadherin (BD: 610182), N-Cadherin (Sigma: C-2542), and Vimentin (Dako: M0725) were used as primary antibodies and anti-Mouse Alexa 488 (Invitrogen: A-11008) was used as secondary antibody. Images were visualized by a Nikon Eclipse e800 microscope and acquired by Nikon ACT-1 software.

### Migration Assay

For transwell migration assay, 24-well PET inserts were used (8.0 μm pore size, Falcon: L003971 F3097) and 4^*^10^4^ cells were seeded. Migrated cells were fixed after 18 h in PFA 4% and stained with Crystal Violet 0.5% (Sigma: C0775). At least 4 images for insert were captured by OLYMPUS CK2 inverted optical microscope at 10× magnification through the digital camera Canon PowerShot A630. Cells were counted with ImageJ software.

### MTS Cell Growth Analysis

2.1^*^10^4^ cells/cm^2^ were seeded in 96 well and every 24 h cell growth was revealed using CellTiter 96® AQueous One Solution Cell Proliferation Assay (Promega: G358C) according to manufacturer's instructions. For detection, at each time point, medium was replaced with a solution composed of 100 μl of PBS/glucose 4.5 g/L (Sigma: G7021) and 20 μl of CellTiter 96® AQueous One Solution in each well.

### Gene Expression Analysis

Total RNA was processed as previously described (26). All RNA samples were checked for genomic contamination via qPCR. qRT-PCR was performed using IQ™ SYBRsGreen Supermix (Bio-Rad: 1708887). The CFX96 Real-Time PCR detection system (Bio-Rad) was used to perform PCR; all the primers (Supplemental Table 1) were designed using Primer3Plus software according to NCBI, Ensembl, and FANTOM-CAT sequence databases. For relative quantification, the GAPDH (Supplemental Table 1) or 18S (38) genes were used as internal standard reference. All experiments were performed at least in duplicate technical replicates. Analyses were done using DDCT method, unless otherwise specified. For classic RT-PCR we used Maxima Hot Start Green PCR master Mix 2X (Thermo Fisher Scientific: FERK1062) and BIOER xp thermal cycler (Genetouch). Amplification products were analyzed on polyacrylamide TBE gel.

### Bioinformatics Analysis

*HMGA1* and *HMGA2 loci* analysis was performed using Zenbu browser genomic data visualization tool from FANTOM-CAT (http://fantom.gsc.riken.jp/cat/). Zenbu was used to visualize transcripts whereas sample ontology association, dynamic expression and genetic trait association coding potential analysis were achieved in FANTOM-CAT Browser (http://fantom.gsc.riken.jp/cat/v1/#/), gene section. For correlation analysis between HMGA2 and HMGA2-AS1 we used Gene Expression Profiling Interactive Analysis (GEPIA) database (39) (http://gepia.cancer-pku.cn/) in BRCA (Breast invasive carcinoma), COAD (Colon adenocarcinoma), LIHC (Liver hepatocellular carcinoma), PAAD (Pancreatic adenocarcinoma), PRAD (Prostate adenocarcinoma), and THAC (Thyroid carcinoma) datasets. Spearman correlation coefficient was employed. For the overall survival analysis, Kaplan–Meier survival analysis of HMGA2 (90-cases) or HMGA2-AS1 (85-cases) was obtained from GEPIA (http://gepia.cancer-pku.cn/) in the PAAD dataset, using quartile (75% cutoff-high, 25% cutoff-low) as group cutoff. For Pathological Stage analysis in PAAD dataset, violin plots in major tumor stages were obtained from GEPIA (http://gepia.cancer-pku.cn/). The method for differential gene expression analysis used was one-way ANOVA.

## Results

### *HMGA1* and *HMGA2 loci* Contain Several Natural Antisense RNAs

**Figure 1:**
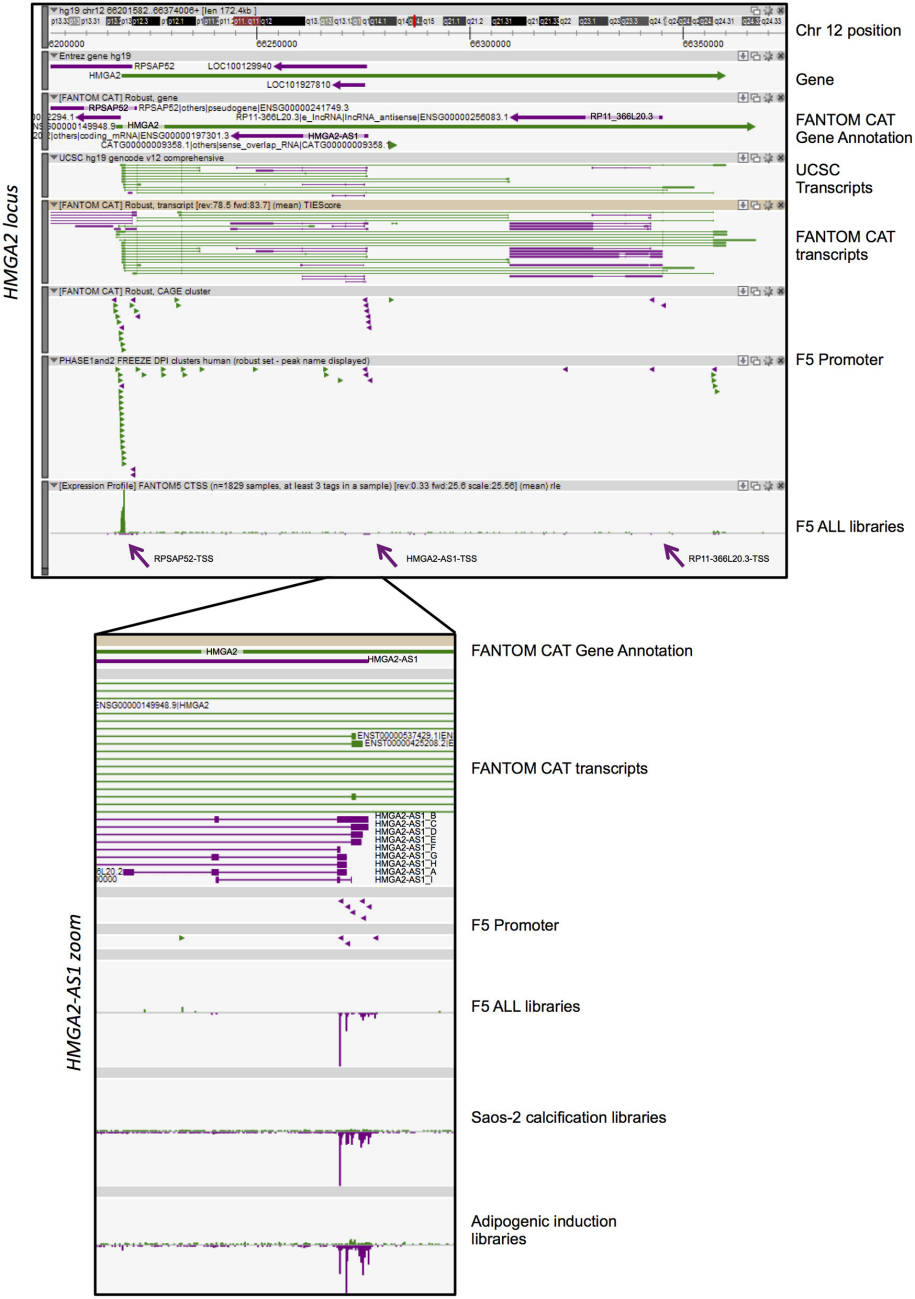
FANTOM-CAT analyses reveal the presence of several natural antisense RNAs in *HMGA2 locus*. A Zenbu genome browser view of gene locus for human *HMGA2*. Genes and transcripts are color-coded according to their orientation in the genome (+ strand, green; – strand, purple). Upper panel reports from top to bottom: Genomic coordinates (Chr 12:66,201,582–66,374,008); NCBI Gene bodies; FANTOM-CAT Gene Annotation. Annotated UCSC transcripts and Robust FANTOM-CAT transcripts, with exon (thick lines) and intron (thin lines) boundaries. FANTOM5 promoters (robust CAT clusters and robust DPI) are indicated as arrowheads. Expression profile visualized as quantitative histogram by FANTOM5 CAGE TSS as the mean of rle (all libraries, *n* = 1,829 samples, at least three tag in a sample). Purple arrows pointed the TSS of RPSAP52, HMGA2-AS1, and RP11-366L20.3. Lower panel contains a zoom of ZENBU visualization of FANTOM-CAT analyses of HMGA2-AS1 natural antisense RNAs that localize in the first part of the HMGA2 third intron. The panel reports from top to bottom: FANTOM-CAT Gene Annotation; Robust FANTOM-CAT transcripts. We report transcript variant name, summarized in Figure 2. FANTOM5 promoters (robust CAT clusters and robust DPI) are indicated as arrowheads. Expression profile is reported as quantitative histogram in all FANTOM5 libraries (rle). Expression profile is shown as quantitative histogram derived from Dynamic expression in Saos-2 calcification and adipogenic induction libraries (tpm).

We used FANTOM5 data resources (40) to investigate antisense transcription in *HMGA1* and *HMGA2 loci*, across 1,829 human samples and identify novel antisense lncRNAs that may have regulatory functions. FANTOM-CAT data visualization in Zenbu (http://fantom.gsc.riken.jp/cat/) of *HMGA1* and *HMGA2 loci* revealed the presence of novel antisense transcripts with consistent Relative log expression (rle) in both *loci* (Supplemental Figure 1 and Figure 1). HMGA1 antisense transcription is concentrated in the promoter region of *HMGA1* where *CATG00000088127.1* gene is located and annotated in FANTOM-CAT as “Promoter-associated lncRNAs” (p_lncRNA_divergent) (Supplemental Figure 1), characterized to be bidirectional transcribed. We analyzed transcriptional start site (TSS) usage from FANTOM5 datasets and observed that CATG00000088127.1 expression is mainly enriched in cells of the hemolymphoid and immuno systems (Supplemental Figure 1, 5′ zoom). Moreover, dynamic expression analysis highlighted the induction of this natural antisense lncRNA in macrophage upon influenza infection (Supplemental Figure 1, 5′ zoom).

*HMGA2 locus* showed a more complex pattern of antisense transcription than *HMGA1*. Indeed, we observed three independent TSS in antisense orientation relative to *HMGA2* transcription, which promoted the transcription of three genes, annotated in FANTOM-CAT as “Other RNAs” (*RPSAP52* and *RP11*-*366L20*.2, also named *HMGA2-AS1*), and “Enhancer lncRNA” (*RP11-366L20.3*) (Figure 1). The first natural antisense gene present in the *HMGA2 locus*, named *RPSAP52* (*ENSG00000241749*), includes a head-to-head divergent to 5′ HMGA2 antisense RNA (Figure 1) and has already been described to be involved in *HMGA2* gene expression regulation (33–35). The second natural antisense gene, originally named *RP11-366L20.2* (*uncharacterized LOC100129940: ENSG00000197301*) and now *HMGA2-AS1* according to HGNC (HUGO Gene Nomenclature Committee), is located in the first part of the *HMGA2* third intron and has not been investigated so far, as well as the third gene, *RP11-366L20.3* (*ENSG00000256083*. *1*), that is localized at the end of the same intron. The highest level of antisense transcription, in terms of positive expression number of libraries, is represented by RPSAP52-TSS (14% of FANTOM5 libraries), with a sum of rle CAGE signal equal to 730. On the contrary, the lowest expression is detected in RP11-366L20.3-TSS (2% of FANTOM5 libraries) with an rle sum of 33.9 (Table 1). RP11-366L20.3 is expressed at a very low level, in quantitative terms, compared to RPSAP52 and HMGA2-AS1. In fact, the highest CAGE signals and the mean of expression underlined that RP11-366L20.3 is poorly expressed (2.6 and 1.0, respectively), in contrast with RPSAP52 and HMGA2-AS1, which have higher and very similar values (Table 1). Considering that RPSAP52 has been already described and RP11-366L20.3 expression was low, we decided to focus on *HMGA2-AS1*.

**Table 1:** Expression parameters of HMGA2, RPSAP52, HMGA2-AS1, and RP11-366L20.3.

**CAGE analysis**	**HMGA2**	**RPSAP52**	**HMGA2-AS1**	**RP11-366L20.3**
Sum of expression (rle)	146,974.2	730.0	221.6	33.9
Positive expression number of libraries (%)	57	14	5	2
Highest signal (rle)	1,553.9	24.2	25.1	2.6
Mean of expression (rle)	140.8	2.9	2.5	1.0

### HMGA2-AS1 Transcript Variants Include Natural Antisense lncRNAs

**Figure 2:**
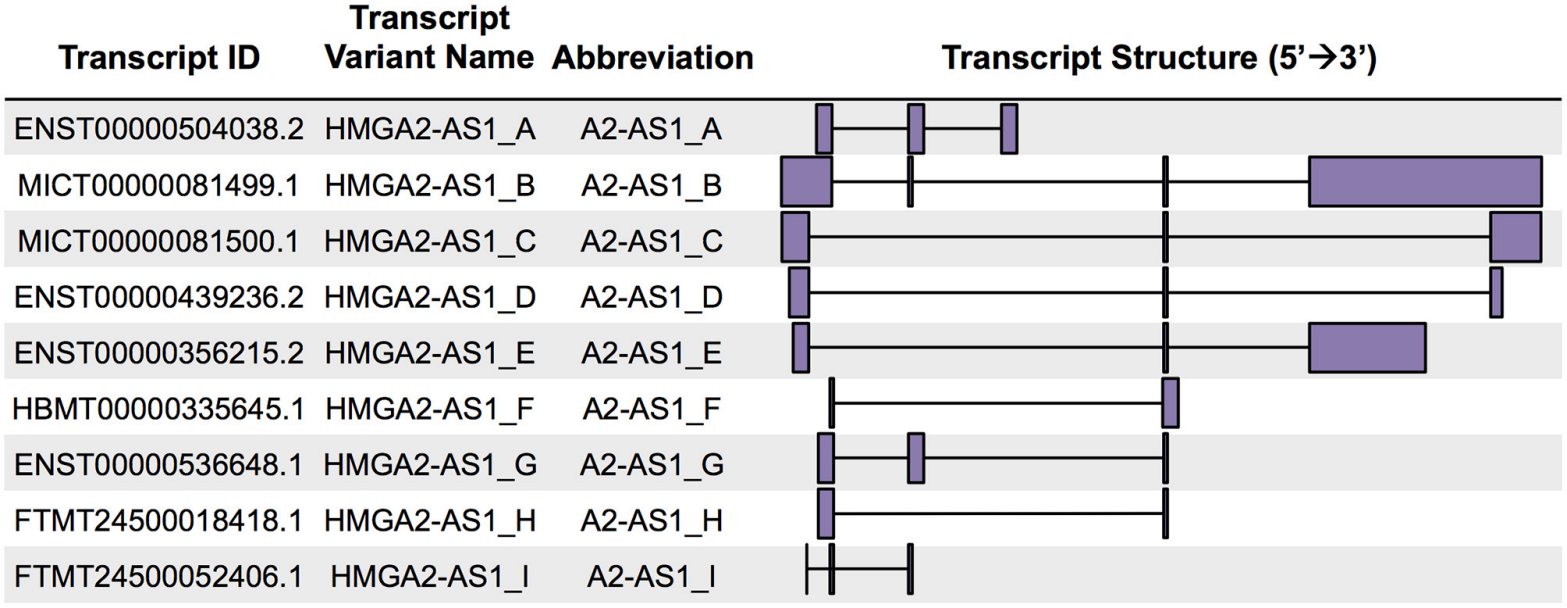
HMGA2-AS1 natural antisense RNAs unraveled by FANTOM-CAT analysis. From left to right: transcript IDs reported in FANTOM-CAT; transcript variant names assigned in this work; abbreviations used throughout the text; and schematic representation (5′ 3′) of the HMGA2-AS1 transcripts.

FANTOM-CAT data visualization in Zenbu of *HMGA2*-*AS1* revealed the presence of nine new transcript variants, not yet annotated in public databases and still uncharacterized. HMGA2-AS1 variants display different exon composition (Figure 1) that, for simplicity, we named from A to I as reported in Figure 2. From robust promoter analysis, we observed that HMGA2-AS1 transcript variants are transcribed from different TSS (Figure 1, HMGA2-AS1 zoom), which could be differentially used in different cell conditions. Indeed, analysis of all FANTOM5 libraries compared to dynamic expression in Saos-2 calcification and adipogenic induction libraries clearly highlighted a different TSS usage (Figure 1, HMGA2-AS1 zoom), suggesting a specific role for each transcript variant in space (cell type) and time (differentiation/response to external stimuli). Notably, in these time course experiments, HMGA2-AS1 is dynamically regulated similarly to HMGA2 (Supplemental Table 2). Moreover, GWAS analysis underline that both *HMGA2* and *HMGA2-AS1* associate with Polycystic Ovary Syndrome and Type 2 Diabetes Mellitus (Supplemental Table 3).

FANTOM-CAT catalog of human genes annotates *HMGA2-AS1* within the category of potentially protein-coding CAT gene class. The same gene is included within lncRNAs in NCBI and ENSEMBL. Given the complexity of transcript variants that we observed within this *locus* and the alternative expression in different cell types and during differentiation/response to external cues, we decided to further deepen this aspect and firstly analyzed the coding potential for each transcript variant individually. We took in consideration different tools based on RNA intrinsic characteristics (cPAT) or on phylogenetic conservation (RNACode, phyloCSF, and sORF ribose). Despite RNACode, phyloCSF and sORF ribose calculated no coding potential for all the transcript variants (Supplemental Table 4), cPAT calculated a significant coding potential for A2-AS1_C, A2-AS1_D, and A2-AS1_E (Supplemental Table 5), identifying A2-AS1_A, A2-AS1_B, A2-AS1_F, A2-AS1_G, A2-AS1_H, and A2-AS1_I as natural antisense lncRNAs. LncRNAs are poorly evolutionary conserved (41), thus tools based on comparative sequence analysis software, such as RNACode, phyloCSF, and sORF ribose could be less informative to predict coding potential than alignment-free programs as cPAT (42). Indeed, we analyzed the evolutionary conservation of *HMGA2-AS1* across 35 mammalian genomes using the EPO Multiple Alignment and we found that *HMGA2-AS1* DNA sequence was strongly and limitedly conserved in primates (Supplemental Figure 2), whereas no conservation was observed in other mammalian species suggesting an importance of *HMGA2-AS1* in this *Order* and supporting the results of cPAT. With these analyses we found a novel locus of natural antisense transcripts in *HMGA2* gene composed by six lncRNAs and three potentially coding transcripts.

### HMGA2-AS1 Transcript Variants Are Expressed in Cancer Cell Lines

**Figure 3:**
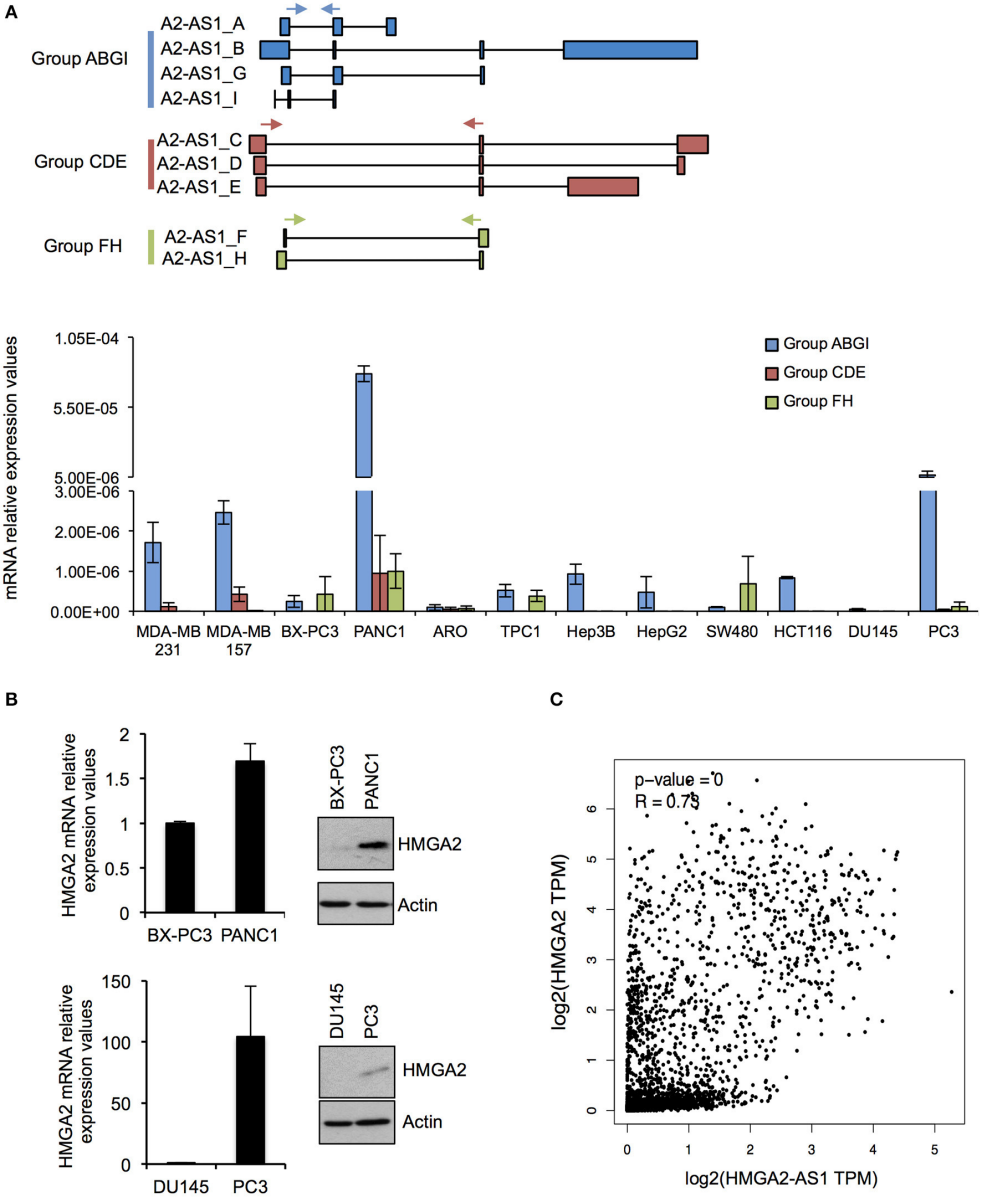
HMGA2-AS1 transcript variants are expressed in cancer cell lines. **(A)** Upper part shows a schematic representation of primer localization (cyan, red and green arrows) used for amplifying HMGA2-AS1 transcript variants, grouped in Group ABGI (cyan), Group CDE (red), and Group FH (green). Lower part shows qRT-PCR analysis of the three transcript groups in a panel of cancer cell lines. 18S was used for normalization. Data are presented as the mean of 2∧–DCt ± range between replicates (*n* = 2). **(B)** qRT-PCR and western blot analyses of HMGA2 in BX-PC3, PANC1, DU145, and PC3 cancer cell lines. For qRT-PCR 18S was used for normalization. Data are presented as the mean ± range between replicates (*n* = 2). For protein analysis a representative western blot is reported. β-actin was used as loading control (*n* = 2). Also see uncropped figure scan in Supplemental Images 1, 2. **(C)** Plot of correlation of HMGA2 and HMGA2-AS1 expression in a TCGA dataset that includes BRCA (Breast invasive carcinoma), COAD (Colon adenocarcinoma), LIHC (Liver hepatocellular carcinoma), PAAD (Pancreatic adenocarcinoma), PRAD (Prostate adenocarcinoma), and THAC (Thyroid carcinoma) datasets. Data were presented in log2 scale and Spearman correlation coefficient was used.

As a first step in characterizing the RNAs present in the *HMGA2-AS1 locus*, we analyzed their expression in human cell lines derived from breast (MDA-MB 231, MDA-MB 157), pancreatic (BX-PC3, PANC1), colon (SW480, HCT116), thyroid (ARO, TPC1), hepatic (HepG2, Hep3B), and prostatic (DU145, PC3) carcinoma. Given the complexity of the *locus* we subdivided HMGA2-AS1 transcripts in three detection-groups (Group ABGI, Group CDE, and Group FH) based on their common exons composition (Figure 3A), and we analyzed their expression by qRT-PCR. Results showed that all the groups of transcripts were expressed in several cell lines although at different levels (Figure 3A). The Group ABGI is the most expressed, whereas the Group CDE, which is composed by potential coding transcript variants, is almost undetectable in most cell lines (Figure 3A). Interestingly, in pancreatic tumors the highest expression of HMGA2-AS1 transcripts was found in PANC1 cell line, which is considered more aggressive than BX-PC3 (43–45). Similarly, we observed a higher expression of HMGA2-AS1 transcripts in prostatic cancer cell line PC3 with respect to DU145 cell line (Figure 3A). In this case PC3 cell line has also a behavior that indicates a more metastatic potential than DU145 cell line, in fact it exhibits a stellate phenotype in 3D culture instead of DU145 that is characterized by a 3D round structure (46). Considering the pro-tumorigenic role of HMGA2 in pancreatic and prostatic cancer (47–50), we checked HMGA2 mRNA and protein levels in these cell lines. Both HMGA2 mRNA and protein are more expressed in PANC1 and PC3 than BX-PC3 and DU145, respectively (Figure 3B) and, interestingly, HMGA2 expression parallels HMGA2-AS1 transcripts expression. Moreover, we observed a significant positive correlation between HMGA2 and HMGA2-AS1 expression in TCGA (The Cancer Genome Atlas) data derived from breast invasive carcinoma, colon adenocarcinoma, liver hepatocellular carcinoma, pancreatic adenocarcinoma, prostate adenocarcinoma, and thyroid carcinoma patient datasets (Figure 3C). Given these results, we reasoned about a possible role of HMGA2-AS1 transcripts in the modulation of HMGA2 expression and tumorigenesis focusing on the non-coding transcript variants (i.e., Group ABGI and Group FH).

### Natural Antisense lncRNAs From *HMGA2-AS1 locus* Regulate HMGA2 Expression

**Figure 4:**
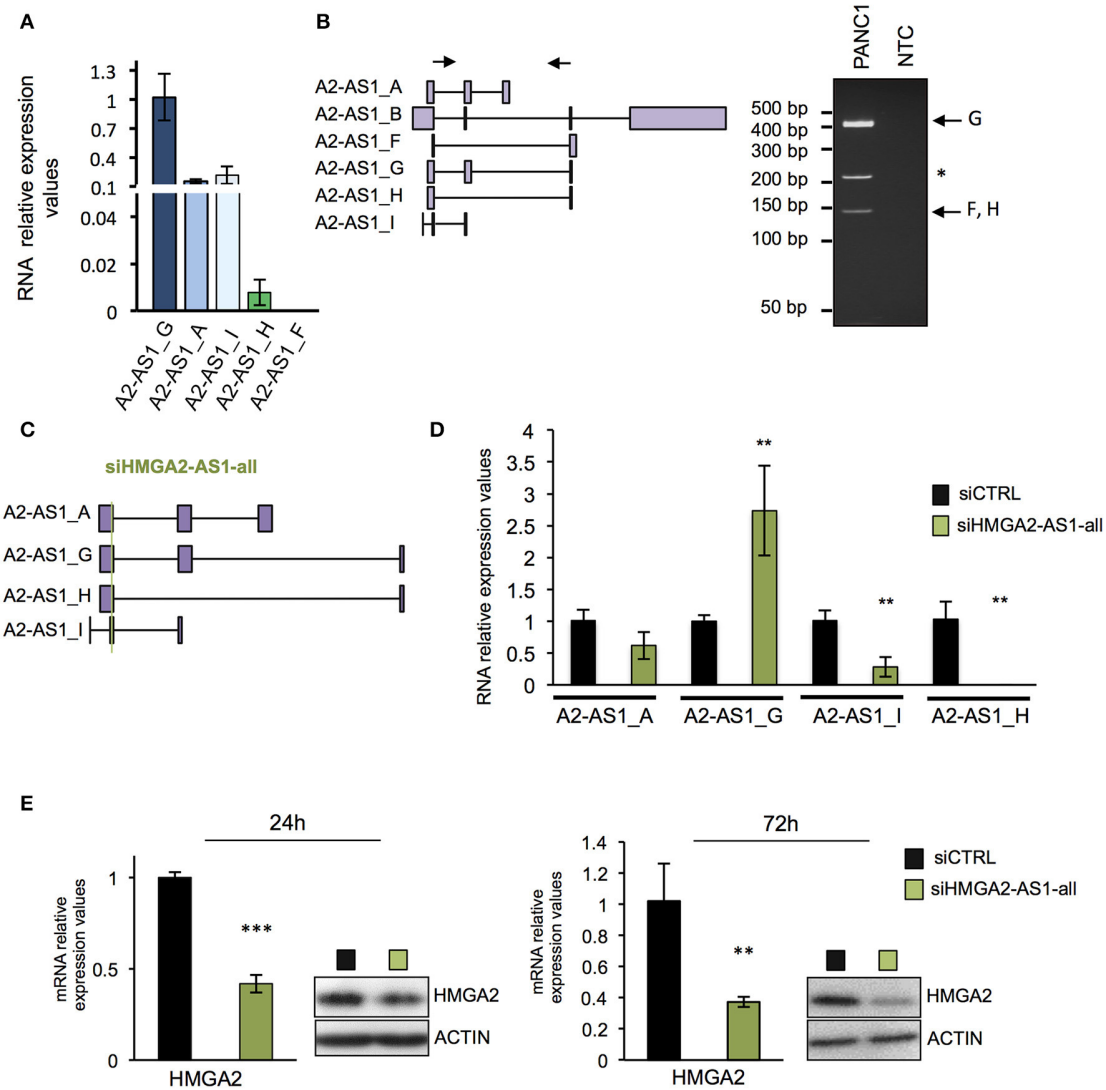
Natural antisense lncRNAs from *HMGA2-AS1 locus* regulate HMGA2 expression. **(A)** qRT-PCR analyses of A2-AS1_G, A2-AS1_A, A2-AS1_I, A2-AS1_H, and A2-AS1_F expression in PANC1 cells. 18S was used for normalization; the data are compared to A2-AS1_G and are presented as the mean ± SD (*n* = 4). **(B)** Left part, schematic representation of primers localization used for amplifying A2-AS1_B, A2-AS1_F, A2-AS1_G, and A2-AS1_H via classical RT-PCR. Right panel shows representative RT-PCR amplification products, * indicates an unspecific product. NTC: no template control. **(C)** Schematic representation of siHMGA2-AS1-all targeting (green line) on each HMGA2-AS1 transcript variants. **(D)** Evaluation of the expression of different variants after siHMGA2-AS1-all transfection. qRT-PCR analysis of A2-AS1_A, A2-AS1_G, A2-AS1_I, and A2-AS1_H levels after 72 h of siHMGA2-AS1-all silencing in PANC1 cell line. 18S was used for normalization. The data are compared to siCTRL and are presented as the mean ± SD (*n* = 3), **p ≤ 0.01; two-tailed Student's *t*-test. **(E)** qRT-PCR and western blot analyses of HMGA2 in PANC1 cells silenced with siHMGA2-AS1-all for 24 and 72 h. For qRT-PCR GAPDH was used for normalization, the data are compared to siCTRL and are presented as the mean ± SD (*n* = 3), **p ≤ 0.01, ***p ≤ 0.001; two-tailed Student's *t*-test. For protein analysis, a representative western blot is reported (*n* = 3). β-actin was used as a loading control. Also see uncropped figure scan in Supplemental Images 3–6.

Many evidences demonstrated that natural antisense lncRNAs could regulate their own sense genes, assuming a crucial role in pathological condition when their expression is impaired (51). We thus investigated whether HMGA2-AS1 natural antisense lncRNAs are involved in HMGA2 expression regulation. Firstly, we analyzed the expression of each transcript variants in PANC1 cell line demonstrating the presence of A2-AS1_G, A2-AS1_A, A2-AS1_I, and A2-AS1_H via qRT-PCR (Figure 4A). Since it was not possible to design suitable primers to analyze specifically A2-AS1_B, we performed classical RT-PCR able to amplify this transcript variant together with A2-AS1_G and A2-AS1_F/H. The amplified products were sequenced, confirming the expression of A2-AS1_G and A2-AS1_F/H and excluding the expression of A2-AS1_B (Figure 4B). Then, we silenced HMGA2-AS1 natural antisense lncRNAs in PANC1 with a small interfering RNA (siRNA) designed to target all transcript variants (siHMGA2-AS1-all) (Figure 4C). We observed a strong reduction of A2-AS1_H and A2-AS1_I amount and a slight decrease of the A2-AS1_A, surprisingly we detected an up-regulation of A2-AS1_G levels (Figure 4D), suggesting no inhibitory action on this transcript variant by siHMGA2-AS1-all. Concomitantly, we highlighted a strong reduction of HMGA2 mRNA and protein levels 72 h after siRNA transfection that was already detectable at 24 h (Figure 4E). Then, we used a second siRNA to confirm the results observed. Unfortunately, it was not possible to design a siRNA in a different region able to target all the four transcript variants analyzed with siHMGA2-AS1-all. Thus, we designed a siRNA, siHMGA2-AS1-AGI, able to target 3 out of the 4 transcript variants, i.e., A2-AS1_A, A2-AS1_G, and A2-AS1_I (Supplemental Figure 3A). We observed the silencing of A2-AS1_I and A2-AS1_A and the up-regulation of A2-AS1_G also with the second siRNA (Supplemental Figure 3B). Moreover, we confirmed the concomitant decrease of HMGA2 levels at 24 and 72 h both for mRNA and protein levels (Supplemental Figure 3C). The down-regulation of HMGA2 upon HMGA2-AS1 silencing, with both siRNAs, was also confirmed in PC3, a prostatic cancer cell line that exhibits high levels of HMGA2-AS1 (Supplemental Figure 4A). Since it was not possible to specifically target the A2-AS1_H with a second siRNA, on a different exon, without hitting A2-AS1_C, A2-AS1_D, and A2-AS1_E, we decided to assess its relevance in regulating HMGA2 expression overexpressing A2-AS1_H in PANC1 cells and we demonstrated that endogenous HMGA2 mRNA expression was up-regulated (Supplemental Figure 4B). As shown above (Figure 4D and Supplemental Figure 3B) upon siRNA treatment against HMGA2-AS1 we observed an unexpected up-regulation of A2-AS1_G. We are not able to explain this modulation, but we tested whether it could regulate HMGA2 expression. Therefore, we overexpressed A2-AS1_G in PANC1 cells and we did not detect any changes in HMGA2 expression levels, demonstrating that A2-AS1_G is not involved in HMGA2 regulation (Supplemental Figure 4C). This data clearly indicates the involvement of HMGA2-AS1 natural antisense lncRNAs, in particular A2-AS1_H, A2-AS1_I, and A2-AS1_A transcript variants, in HMGA2 gene expression regulation.

### HMGA2-AS1 lncRNAs Are Involved in Cancer Promotion

**Figure 5:**
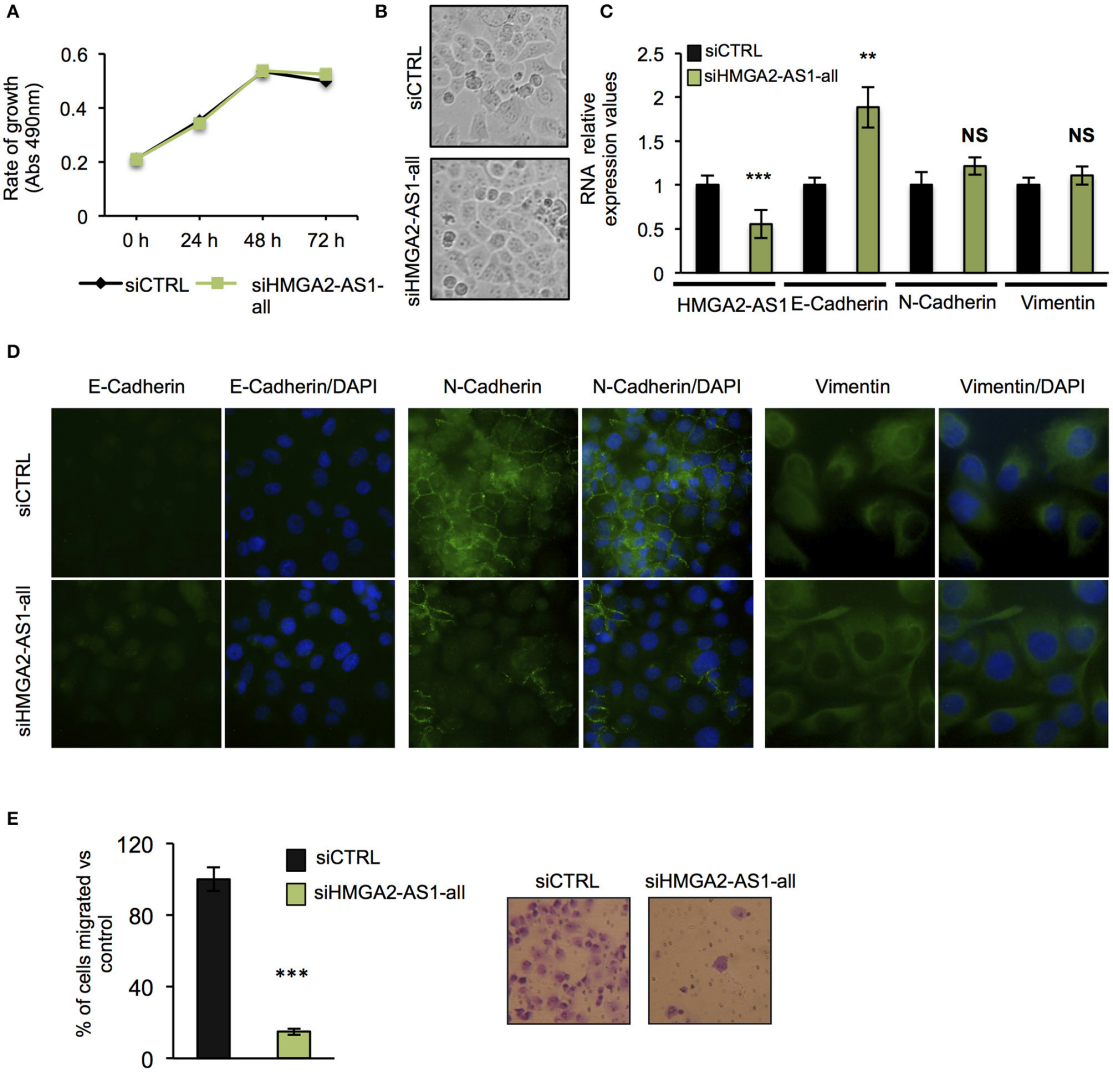
Natural antisense lncRNAs from *HMGA2-AS1 locus* are involved in cancer promotion. **(A)** MTS proliferation assay in PANC1 cells silenced or not for HMGA2-AS1 transcript variants with siHMGA2-AS1-all. The data are presented as mean ± SD (*n* = 4). **(B)** Representative pictures of cell morphology of PANC1 cell culture in control condition and after 72 h of siHMGA2-AS1-all transfection. **(C)** qRT-PCR analysis of HMGA2-AS1, E-Cadherin, N-Cadherin, and Vimentin after 72 h of siHMGA2-AS1-all silencing in PANC1 cell line. Primers used to detect HMGA2-AS1 amplify together A2-AS1_A, A2-AS1_G, and A2-AS1_I. GAPDH was used for normalization. The data are compared to siCTRL and are presented as the mean ± SD (*n* = 3), **p ≤ 0.01, ***p ≤ 0.001; two-tailed Student's *t-*test. **(D)** Representative immunofluorescence images of the epithelial marker E-Cadherin and the mesenchymal markers N-Cadherin and Vimentin localization in PANC1 control cells vs. cells silenced with siHMGA2-AS1-all. Images were taken at 40× magnification for E- and N-Cadherin and at 60× magnification for Vimentin. **(E)** Transwell assay of PANC1 cells silenced with siHMGA2-AS1-all for 72 h. On the left, quantification of the transwell assay. The data are represented as the mean of percentage of migrated cells respect to siCTRL ± SD (*n* = 4), ***p ≤ 0.001; two-tailed Student's *t*-test. On the right, representative images of cells migrated across the porous membrane, stained with crystal violet.

**Figure 6:**
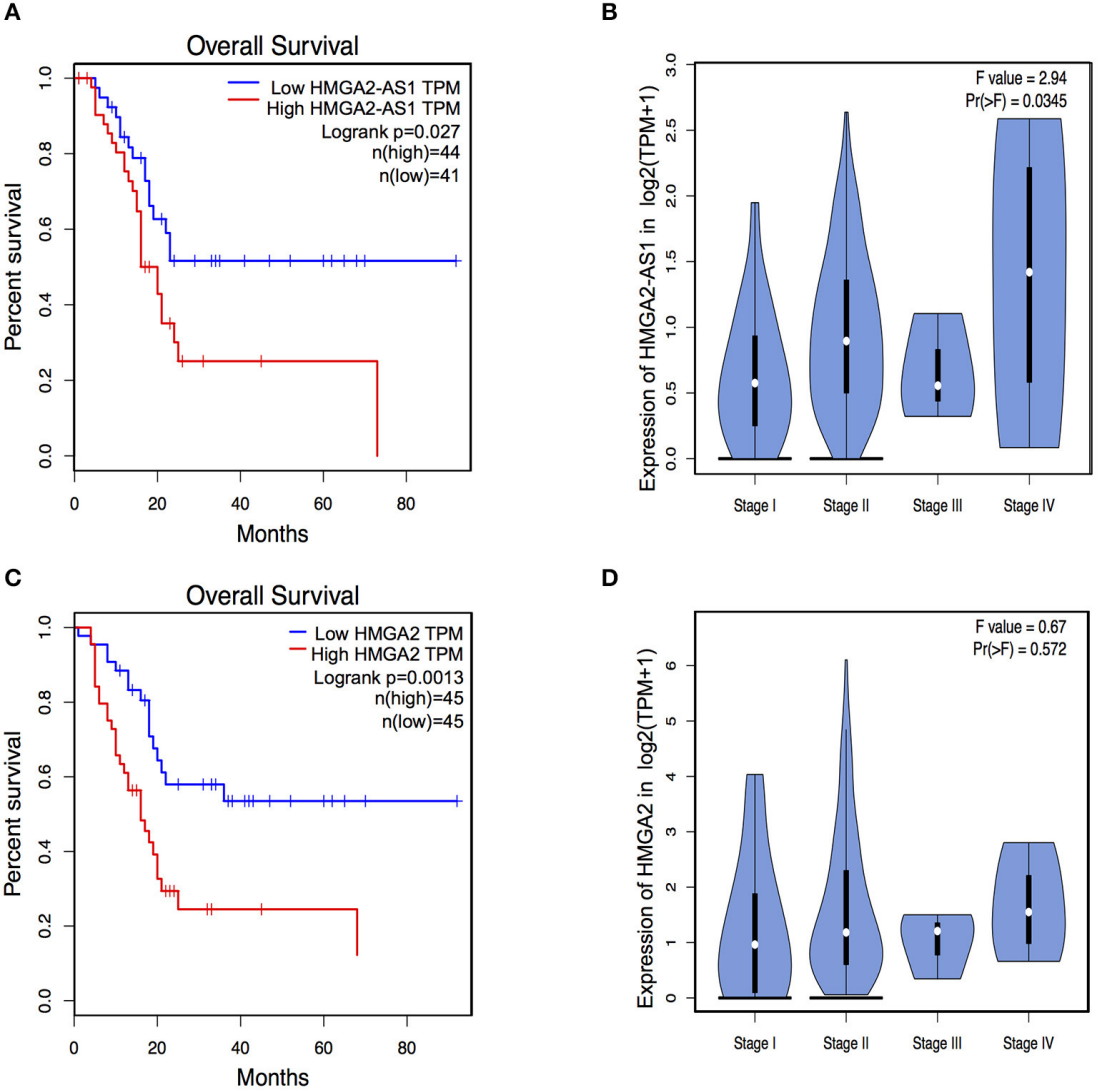
HMGA2-AS1 and HMGA2 expression is relevant for Overall Survival (OS) in pancreatic cancer patients. **(A)** Kaplan–Meier survival curves of OS in a dataset of pancreatic adenocarcinomas patients. The patients were stratified based on the expression of HMGA2-AS1. **(B)** Violin plot of HMGA2-AS1 enrichment expression in pancreatic adenocarcinomas patients from PAAD dataset, subdivided accordingly to cancer stage. **(C)** Kaplan–Meier survival curves of OS in a dataset of pancreatic adenocarcinomas patients. The patients were stratified based on the expression of HMGA2. **(D)** Violin plot of HMGA2 enrichment expression in pancreatic adenocarcinomas patients from PAAD dataset, subdivided accordingly to cancer stage.

The role of several lncRNAs in cancer onset and progression has been demonstrated (52), underlying that alteration in their expression could be crucial in this disease. Moreover, the involvement of HMGA2 in promoting cancer hallmarks connected with the tumorigenic processes is widely described (20, 28, 53). Therefore, we asked whether changes in the expression of HMGA2-AS1 natural antisense lncRNAs may have a role in the tumorigenic process, in particular we started analyzing cell proliferation. PANC1 cells were silenced (siHMGA2-AS1-all) or not (siCTRL) for the expression of HMGA2-AS1 natural antisense lncRNAs and cell growth was analyzed at different time points (24, 48, and 72 h). No difference in cell growth was observed in silenced with respect to control cells (Figure 5A). Despite PANC1 cells showed some epithelial features (54), upon HMGA2-AS1 silencing these characteristics were exacerbated. Indeed, cells were flatter exhibiting a cobblestone shape and cell culture appeared more organized (Figure 5B). In addition, we observed an increase of the epithelial marker E-Cadherin (Figures 5C,D). We analyzed also two mesenchymal markers, N-Cadherin and Vimentin, and while we did not observe changes at the RNA level (Figure 5C) we found a delocalization of N-Cadherin from cell membrane and a decreased perinuclear density of Vimentin (Figure 5D), which is connected to a decrease in cell motility (55). On the basis of these results and considering the involvement of HMGA2 in cell migration (24, 48, 56), we tested whether HMGA2-AS1 natural antisense lncRNAs were involved in this key tumor feature. Thus, we analyzed cell motility by transwell assay after siHMGA2-AS1-all treatment in PANC1 cells, highlighting a strong decrease in the ability of cells to move across the membrane pore (Figure 5E), suggesting an involvement of HMGA2-AS1 in metastatic process. All these results were confirmed silencing HMGA2-AS1 using the second siHMGA2-AS1-AGI (Supplemental Figures 5A–D). Moreover, we demonstrated the involvement of HMGA2-AS1 in cancer cell motility using PC3 cell line silenced with both siRNAs (Supplemental Figure 6A) and overexpressing A2-AS1_H transcript variant in BX-PC3 (Supplemental Figures 6B,C), a pancreatic cell line that we showed expressing low level of all HMGA2-AS1 transcript variants (see Figure 3A). Taking into account these results, we explored the relationship between HMGA2-AS1 and the prognosis of pancreatic adenocarcinoma patients in terms of overall-survival (OS). Kaplan-Meier analysis shows that a higher HMGA2-AS1 expression was associated with a shorter OS (*P* = 0.03) (Figure 6A). In addition, we observed in the same dataset an enrichment of HMGA2-AS1 expression in pathological Stage IV (*Pr* ≥ 0.035) (Figure 6B). All these data clearly suggest a tumorigenesis function of HMGA2-AS1 in pancreatic cancer.

### HMGA2-AS1 lncRNAs Regulate Cell Migration Ability Through HMGA2

**Figure 7:**
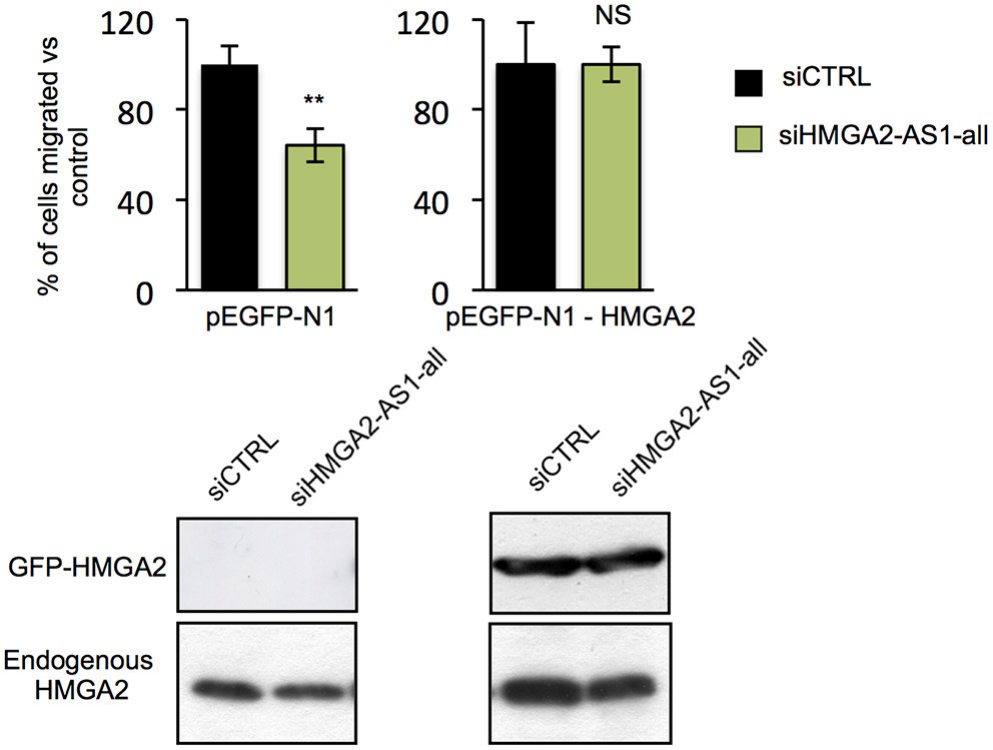
HMGA2-AS1 lncRNAs regulate cell migration ability through HMGA2. Transwell assay in PANC1 cells transfected with empty vector (pEGFP-N1) or with a vector expressing HMGA2 (pEGFP-N1-HMGA2), treated with siCTRL or siHMGA2-AS1-all. The data are presented in pEGFP-N1 and in pEGFP-N1-HMGA2 conditions as the mean of the percentage of siHMGA2-AS1-all migrated cells relative to the siCTRL ± SD (*n* = 3) set to 100% in each condition. Lower part, a representative western blot of HMGA2 overexpression and endogenous protein is presented. β-actin was used as a loading control. **p ≤ 0.01, NS: Not Significant; two-tailed Student's *t*-test. Also see uncropped figure scan in Supplemental Images 7–9.

HMGA2 protein has a relevant and causal role in cancer onset and development, supporting metastatic process and its involvement in pancreatic cancer has been already described (48, 57). Exploring the relationship between HMGA2 and OS of pancreatic adenocarcinoma patients, we observed that higher HMGA2 expression was associated with a shorter OS (*P* = 0.0013) (Figure 6C), similarly to what observed for HMGA2-AS1 (Figure 6A), in addition, a trend in the increase of expression of HMGA2 through the different stages was found (Figure 6D). Our results show that natural antisense lncRNAs HMGA2-AS1 modulate motility of PANC1 cells and they regulate HMGA2 expression. We therefore asked whether the effect of HMGA2-AS1 on cell motility was mediated by HMGA2. To this aim, we silenced the expression of HMGA2-AS1 (siHMGA2-AS1-all) and we overexpressed HMGA2 (pEGFP-N1-HMGA2) to assess whether HMGA2 was able to rescue PANC1 cell migration abilities. Whereas PANC1 cells depleted for HMGA2-AS1 showed a strong decrease in cell migration compared to control, the overexpression of HMGA2 was able to completely rescue cell migration (Figure 7), demonstrating that HMGA2-AS1 are important players in tumorigenesis and that this function is mediated by HMGA2.

## Discussion

Deregulation of HMGA proteins in adult tissue is strictly associated with neoplastic transformation, in fact high expression levels of these proteins have been found in several types of tumor (19, 20, 58). Therefore, the fine modulation of their expression is crucial and several literature data underline that HMGA expression is controlled at different regulatory levels, from transcriptional to post-translational, and by several players (28, 59, 60). Remarkably, over the past decade, a large number of non-coding RNA molecules have been found to belong to the HMGA-expression control network (32, 60, 61). A key step derives from regulation operated by miRNA, considering that both HMGA1 and HMGA2 are targets of the tumor suppressor let-7 (62). However, the comprehension of HMGA expression regulation is still far from being completely understood.

Natural antisense lncRNAs are often expressed from cancer-associated gene *loci* together with the concordant expression of their own sense genes (63). In this context, antisense transcription is increasingly being recognized as a crucial regulator of sense gene expression in response to pathological stimuli. Therefore, with the aim to investigate the presence of sense and antisense transcripts pairing (S/AS pairs) in *HMGA loci* and the possible control of *HMGA* expression by antisense lncRNAs, we interrogated the FANTOM5 and FANTOM-CAT catalogs. FANTOM5 project enormously increased the number of ncRNA annotated, especially lncRNA, generating a comprehensive atlas of 27,919 human lncRNA genes (40). Now, a huge effort is required to understand the function of these lncRNAs. Indeed, recently, it has been demonstrated the relevance of antisense transcription in *loci* associated with hereditary neurodegenerative disease, providing evidences for the existence of additional regulatory mechanisms of the expression of neurodegenerative disease-causing genes (64).

Here, we show a complex picture of antisense transcription in *HMGA2* gene, increasing the number of molecules possibly involved in HMGA2 expression regulation, while *HMGA1 locus* exhibited a lower antisense transcription. Among antisense transcription genes in *HMGA2 locus*, we have found the previously characterized head-to-head natural antisense lncRNA RPSAP52 (33–35). In this study, in addition to RPSAP52, we provide, for the first time to our knowledge, evidences for the existence of previously unknown natural antisense lncRNAs within *HMGA2* gene with a function in HMGA2 expression regulation and neoplastic transformation. Indeed, our analyses on FANTOM-CAT data revealed robust antisense transcriptional activity concentrated in the third intron of *HMGA2* gene and several uncharacterized transcript variants (HMGA2-AS1_A-I) associated. Dynamic expression analysis of FANTOM5 samples showed that transcription of *HMGA2-AS1* gene is significantly up-regulated during mesenchymal stem cells differentiation to adipocyte and down-regulated throughout Saos-2 calcification similarly to what happens for *HMGA2*, suggesting a coordinated role of both genes in these processes. Notably, this observation fits very well with the well-studied role of *HMGA2* in adipogenesis and osteogenesis (65–67).

We demonstrated that some HMGA2-AS1 variants are expressed in different cancer cell lines, in particular in cells from pancreatic adenocarcinoma. Moreover, we found that HMGA2-AS1 positively correlated with HMGA2 expression in a TCGA dataset of cancer patients, and, in *in vitro* experiments, we demonstrated that HMGA2-AS1 increased HMGA2 expression. Given the relevance of fine regulation of HMGA2 expression for a normal development and a correct tissue homeostasis and considering the role of natural antisense lncRNAs in sense-gene expression regulation, the identification of these novel natural antisense lncRNAs can have significant implications in studying cancer pathogenesis. Interestingly, we found that HMGA2-AS1 promoted changes in the expression and localization of markers involved in cell-cell adhesion that support the HMGA2-mediated modulation of cell motility observed in PANC1 cells. These *in vitro* observations of the role of HMGA2-AS1 in promoting pancreatic neoplastic transformation are further reinforced by primary tumor data, showing that HMGA2-AS1 is enriched in patients with high-grade pancreatic adenocarcinoma and its high expression level correlated with poor prognosis in cancer patients.

It is still an open question how HMGA2-AS1 can regulate HMGA2 expression. Natural antisense lncRNAs can modulate their own sense gene expression at multiple levels (68). Indeed, these molecules can regulate the transcription of sense genes by controlling the epigenetic state (69–71), by forming DNA:RNA hybrids (33) or by competing for the same promoter (68, 72, 73). S/AS pairs, instead, mainly mediate post-transcriptional and translational regulation. In fact, S/AS pairs regulate RNA maturation and stability by establishing a physical obstruction to regulatory factors that induces splicing (74) or by influencing RNA stability (63, 75–77). At translational level, antisense transcript lncRNAs can compete with sense RNA for translation initiation factor (78) or induce translation by 5′UTR sense RNA binding (38, 79). Notably, using the RNAup package (80, 81), we observed a 16 nucleotides region of hybridization, localized in the 5′UTR of HMGA2 and in the common exon of natural antisense lncRNAs transcribed by *HMGA2-AS1 locus*, suggesting the existence of a possible HMGA2-AS1:HMGA2 mRNA interaction. Further studies will be needed to clarify if HMGA2-AS1 regulates HMGA2 expression through S/AS pairs.

In conclusion, the present study adds a further level of complexity to the regulation of HMGA2 expression in cancer and, considering the huge amount of data derived from the high-throughput sequencing era, it contributes to increase our knowledge of the function of lncRNAs in regulating cellular functions.

## Data Availability Statement

Publicly available datasets were analyzed in this study. This data can be found here: http://fantom.gsc.riken.jp/cat/, http://gepia.cancer-pku.cn/.

## Author Contributions

GR performed most of the experiments. GR and SP analyzed the data. PD performed the experiments on the function of HMGA2-AS1 in cancer cells. GR, SP, SZ, GM, RS, and SG provided the intellectual input and revised the manuscript. GR, SP, and SZ conceptualized and designed the study. GM and SP supervised the study. GR, SP, and GM wrote the manuscript. All authors read and approved the final version of this manuscript.

## Dedication

This work was dedicated to the memory of Silvia Zucchelli, who supervised the research and enthusiastically contributed to the data discussion and interpretation of the results.

### Conflict of Interest

The authors declare that the research was conducted in the absence of any commercial or financial relationships that could be construed as a potential conflict of interest.

## Supplementary Material

Supplemental Images 1–9 Full scans of western blots.
