# Supplementary material for: HMGA2 Antisense Long Non-coding RNAs as New Players in the Regulation of HMGA2 Expression and Pancreatic Cancer Promotion
Source: Front Oncol. 2020 Jan 17;9:1526. doi: 10.3389/fonc.2019.01526 (PMC6978849; doi:10.3389/fonc.2019.01526)
Supplement: Supplementary file 1 [file Data_Sheet_1.pdf]

Supplemental Table 1. Primers used for qRT-PCR and classic RT-PCR.

Target	Primer For	Primer Rev
GAPDH	TCTCTGCTCCTCCTGTTC	GCCCAATACGACCAAATCT
HMGA2	AGCAGAAGCCACTGGAGAAA	TCTTCGGCAGACTCTTGTGA
Group ABGI or HMGA2-AS1	GAGGAGCTGCTGAGTTCAAACA	CTGGGTTGCTGGGTGCTTT
Group CDE	CGGGCTCTTCATTAGCAACC	TGCTGCTGTTTTCCTTCTGCTG
Group FH	GCCAACA TGACACCAAACTCTC	TGCTGCTGTTTTCCTTCTGCTG
A2-AS1_ A	CTCACCTGTCACTCCTCCACTG	TATTGTTGACACCAGCTTGTTGGG
A2-AS1_ F	GCCAACATGACACCAAACTCTC	CTTCTTTTCAGCAGTGCC
A2-AS1_ G	CTCACCTGTCACTCCTCCACTG	TGCTGCTGTTTTCCTTCTGCTG
A2-AS1_ H	CGTGATTAACCCAAGGATGTTG	GCCTTCCCATCTTGACACTCAG
A2-AS1_ I	GGAATGTGTGCGGAGTCAATAC	TCTCTGAGGGTCTCCAGAGTTT
E-Cadherin	AAGTGTCCGAGGACTTTGGCGTG G	CAGCCAGTTGGCAGTGTCTCTCCA
N-Cadherin	CATCACAGTGGCAGCTGGACTTG	GGCCGTGGCTGTGTTTGAAAGG
Vimentin	CCAGCTAACCAACGACAAAGCCC G	TGCGTTCAAGGTCAAGACGTGCCA
A2-AS1_ B/F/G/H classic RT-PCR	GCCAACATGACACCAAACTCTC	TGCTGCTGTTTTCCTTCTGCTG

Supplemental Figure 1

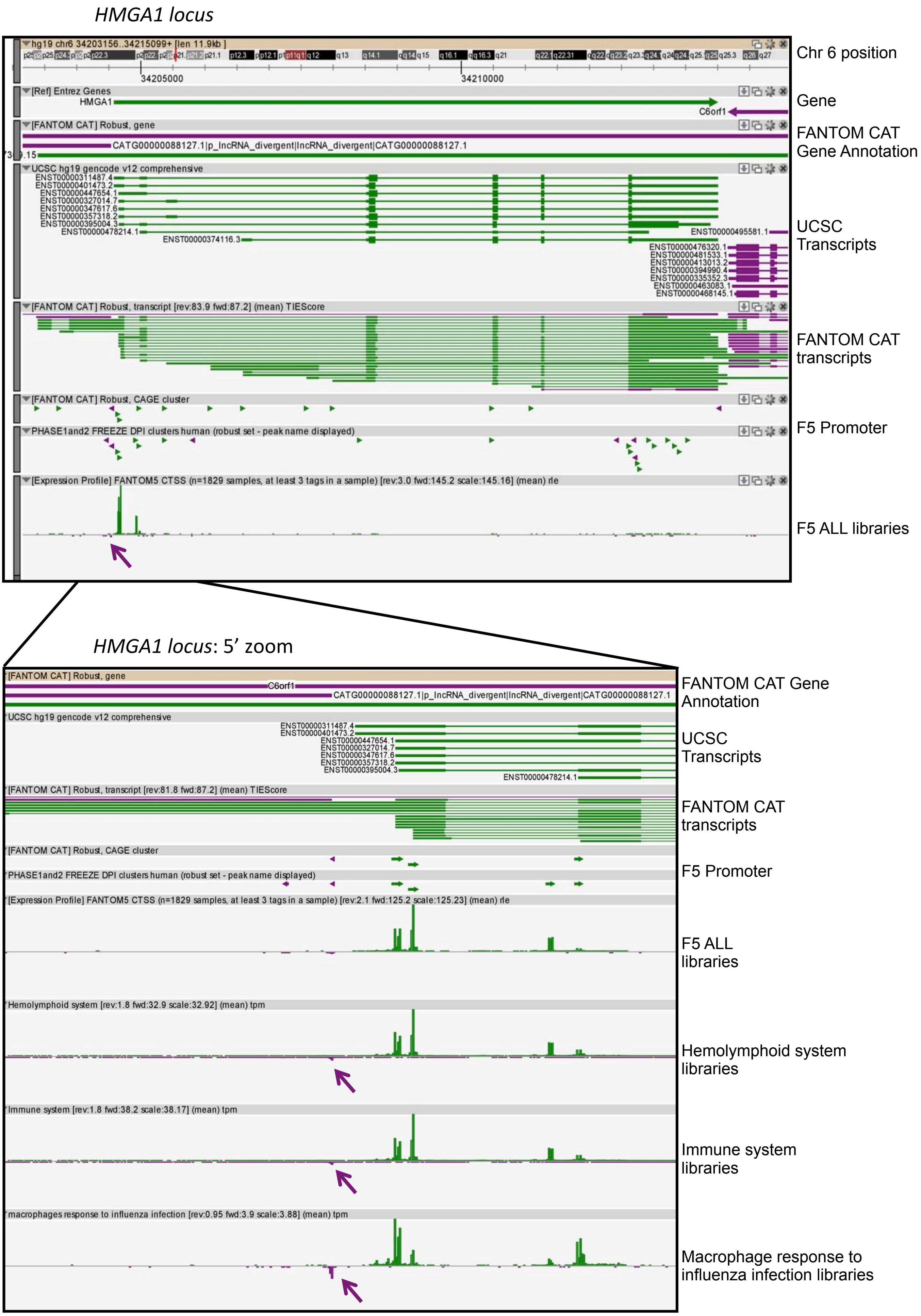

Supplemental Figure 1. FANTOM-CAT analyses reveal the presence of one natural antisense lncRNA in *HMGA1 locus*. A Zenbu genome browser view of gene locus for human *HMGA1*. Genes and transcripts are color-coded according to their orientation in the genome (+ strand, green; − strand, purple). Upper panel reports from top to bottom: Genomic coordinates (Chr 6:34,203,156-34,215,099). NCBI Gene bodies. FANTOM-CAT Gene Annotation. Annotated UCSC transcripts and Robust FANTOM-CAT transcripts, with exon (thick lines) and intron (thin lines) boundaries. FANTOM5 promoters (robust CAT clusters and robust DPI) are indicated as arrowheads. Expression profile is visualized as quantitative histogram by FANTOM5 TSS as the mean of rle (all libraries, n=1829 samples, at least 3 tag in a sample). Lower panel contains a zoom of ZENBU visualization of FANTOM-CAT analyses of *CATG00000088127*.1 that localized in the promoter region of *HMGA1*. The panel reports from top to bottom: FANTOM-CAT Gene Annotation; Annotated UCSC transcripts and Robust FANTOM-CAT transcripts; FANTOM5 promoters (robust CAT clusters and robust DPI) are indicated as arrowheads. Expression profile is reported as quantitative histogram in all FANTOM5 libraries (rle). Expression profile is reported as quantitative histogram derived from Hemolymphoid and Immune system libraries and from Dynamic expression in Macrophage response to influenza infection libraries (tpm). Purple arrow points to the TSS of *CATG00000088127*.1.

Supplemental Table 2. Dynamic Expression of HMGA2, RPSAP52, HMGA2-AS1 and RP11-366L20.3

Gene Name	Series Name	Max FC	FDR
**HMGA2**	mesenchymal stem cells differentiation to adipocyte	-5.56	0.00102
	Myoblast differentiation to myotubes, Duchenne Muscular Dystrophy	-3.34	0.0013
	preadipocyte maturation	-3.23	0.0151
	ARPE-19 EMT induced with TGFb and TNFa, to mesenchymal	1.97	0.000264
	Myoblast differentiation to myotubes, Duchenne Muscular Dystrophy	-3.15	1.87E-09
	293SLAM rinderpest infection	-2.39	1.44E-75
	iPSC differentiation to neuron, Down syndrome	-0.63	0.000483
	Saos-2 osteosarcoma treated with ascorbic acid and BGP to induce calcification	0.99	0.00663
	ARPE-19 EMT induced with TGFb and TNFa, to mesenchymal	1.1	0.00377
	HES3-GFP Embryonic Stem cells cardiomyocytic induction	1.3	0.000073
	H9 Embryoid body cells, melanocytic induction	1.48	0.00172
	COBL-a rinderpest infection	2.48	0.0322
**HMGA2-AS1**	mesenchymal stem cells differentiation to adipocyte	-7.29	9.25E-10
	Saos-2 osteosarcoma treated with ascorbic acid and BGP to induce calcification	2.48	0.00000346

Supplemental Table 3. GWAS of HMGA2, RPSAP52, HMGA2-AS1 and RP11-366L20.3

Gene Name	Trait Name	# of SNPs	Best SNP P-value
**HMGA2**	Polycystic Ovary Syndrome	4	2E-21
	Type 2 Diabetes Mellitus	17	1E-19
	Abnormality Of Body Weight	6	1E-19
	End Stage Renal Failure	6	1E-19
	Birth Weight	6	1E-19
	Alzheimer S Disease	12	1E-12
	Tooth Disease	12	2E-12
	Premature Eruption Of Permanent Teeth	12	2E-12
	Dentition	4	6E-11
	Anodontia	4	1E-10
	Conduct Disorder	6	3E-10
	Neurodegenerative Disease	6	3E-10
	Cognitive Disorder	6	3E-10
	Obesity	6	0.000000222
	Adiposity	6	0.000000222
**HMGA2-AS1**	Polycystic Ovary Syndrome	12	2E-21
	Type 2 Diabetes Mellitus	19	0.000000003

Supplemental Table 4. Coding potential of natural antisense RNAs in the HMGA2-AS1 locus based on cPAT, RNACode, phyloCSF and sORF riboseq tools.

Transcript Variant	Transcript ID	cPAT coding	RNACode coding	phyloCSF coding	sORF riboseq coding
A2-AS1_A	ENST00000504038.2	no	no	no	no
A2-AS1_B	MICT00000081499.1	no	no	no	no
A2-AS1_C	MICT00000081500.1	yes	no	no	no
A2-AS1_D	ENST00000439236.2	yes	no	no	no
A2-AS1_E	ENST00000356215.2	yes	no	no	no
A2-AS1_F	HBMT00000335645.1	no	no	no	no
A2-AS1_G	ENST00000536648.1	no	no	no	no
A2-AS1_H	FTMT24500018418.1	no	no	no	no
A2-AS1_I	FTMT24500052406.1	no	no	no	no

Supplemental Table 5. Parameters of coding potential calculated by cPAT.

Transcript Variant	cPAT Coding	cPAT Coding (Value)	ORF length	RNA length
A2-AS1_A	No	0.28243	210	975
A2-AS1_B	No	0.10705	507	11499
A2-AS1_C	Yes	0.41612	447	2436
A2-AS1_D	Yes	0.43701	447	878
A2-AS1_E	Yes	0.46867	480	4661
A2-AS1_F	No	0.20503	168	691
A2-AS1_G	No	0.28495	210	748
A2-AS1_H	No	0.10034	153	490
A2-AS1_I	No	0.36178	147	246

Supplemental Figure 2

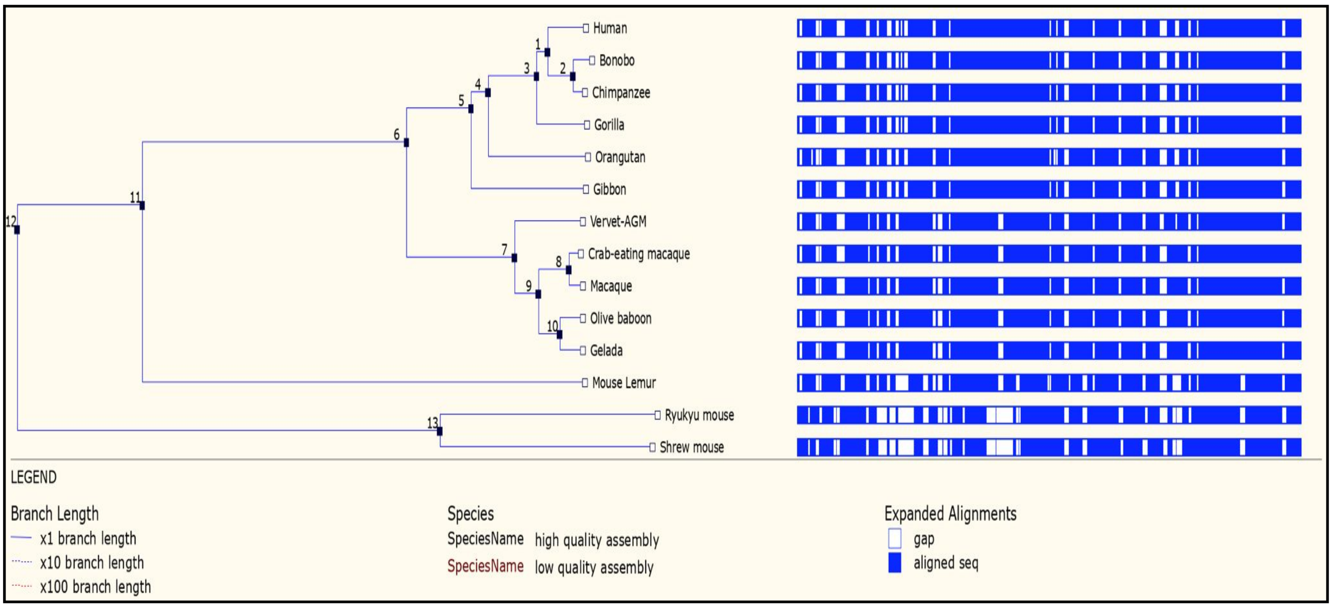

Supplemental Figure 2. Multiple alignment analysis of HMGA2-AS1. EPO (Enredo, Pecan, Ortheus) multiple alignment (Location: 12:65854979_65882767) of 35 mammalian genomes shows conservation of HMGA2-AS1 in primates. No alignment in this region was found for *Mus spretus, Marmota marmota, Cavia aperea, Felis catus, Cricetulus griseus, Bos taurus, Canis lupus dingo, Canis lupus familiaris, Loxodonta africana, Capra hircus, Cavia porcellus, Equus caballus, Panthera pardus, Callithrix jacchus, Mus musculus, Maniculatus bairdii, Sus scrofa, Microtus ochrogaster, Oryctolagus cuniculus, Rattus norvegicus,* and *Ovis aries.*

Supplemental Figure 3

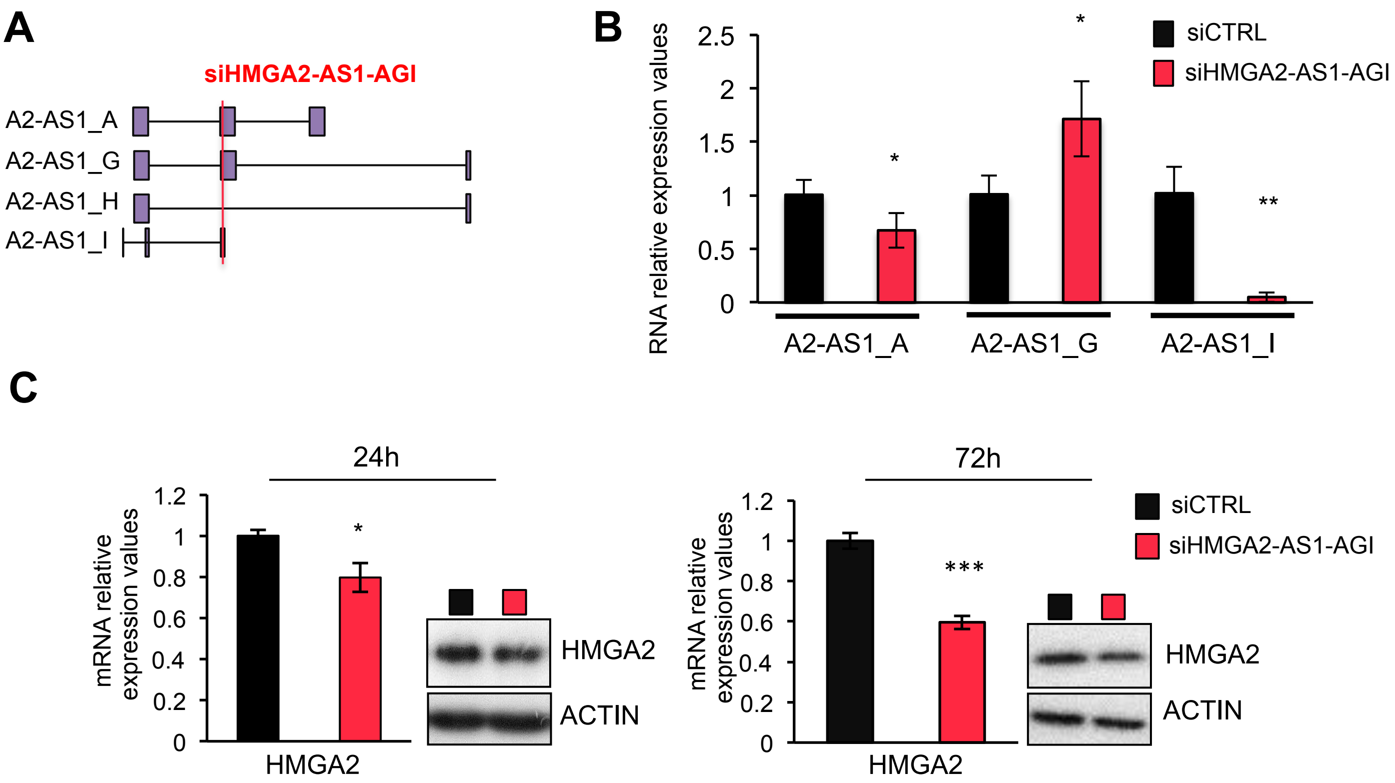

Supplemental Figure 3. Natural antisense lncRNAs from *HMGA2-AS1 locus* regulate HMGA2 expression. (A) Schematic representation of siHMGA2-AS1-AGI targeting (red line) on HMGA2-AS1 transcript variants. **(B)** Evaluation of the expression of different variants after siHMGA2-AS1-AGI transfection. qRT-PCR analysis of A2-AS1_A, A2-AS1_G and A2-AS1_I levels after 72 hours of siHMGA2-AS1-AGI silencing in PANC1 cell line. 18S was used for normalization. The data are compared to siCTRL and are presented as the mean±SD (n=3), *$p \leq 0.05$, **$p \leq 0.01$; two-tailed Student's t-test. **(C)** qRT-PCR and western blot analyses of HMGA2 in PANC1 cells silenced with siHMGA2-AS1-AGI for 24 and 72 hours. For qRT-PCR GAPDH was used for normalization, the data are compared to siCTRL and are presented as the mean±SD (n=3), *$p \leq 0.05$, ***$p \leq 0.001$; two-tailed Student's t-test. For protein analysis, a representative western blot is reported (n=3). β-actin was used as a loading control. Also see uncropped figure scan in Supplementary Image 3-6.

Supplemental Figure 4

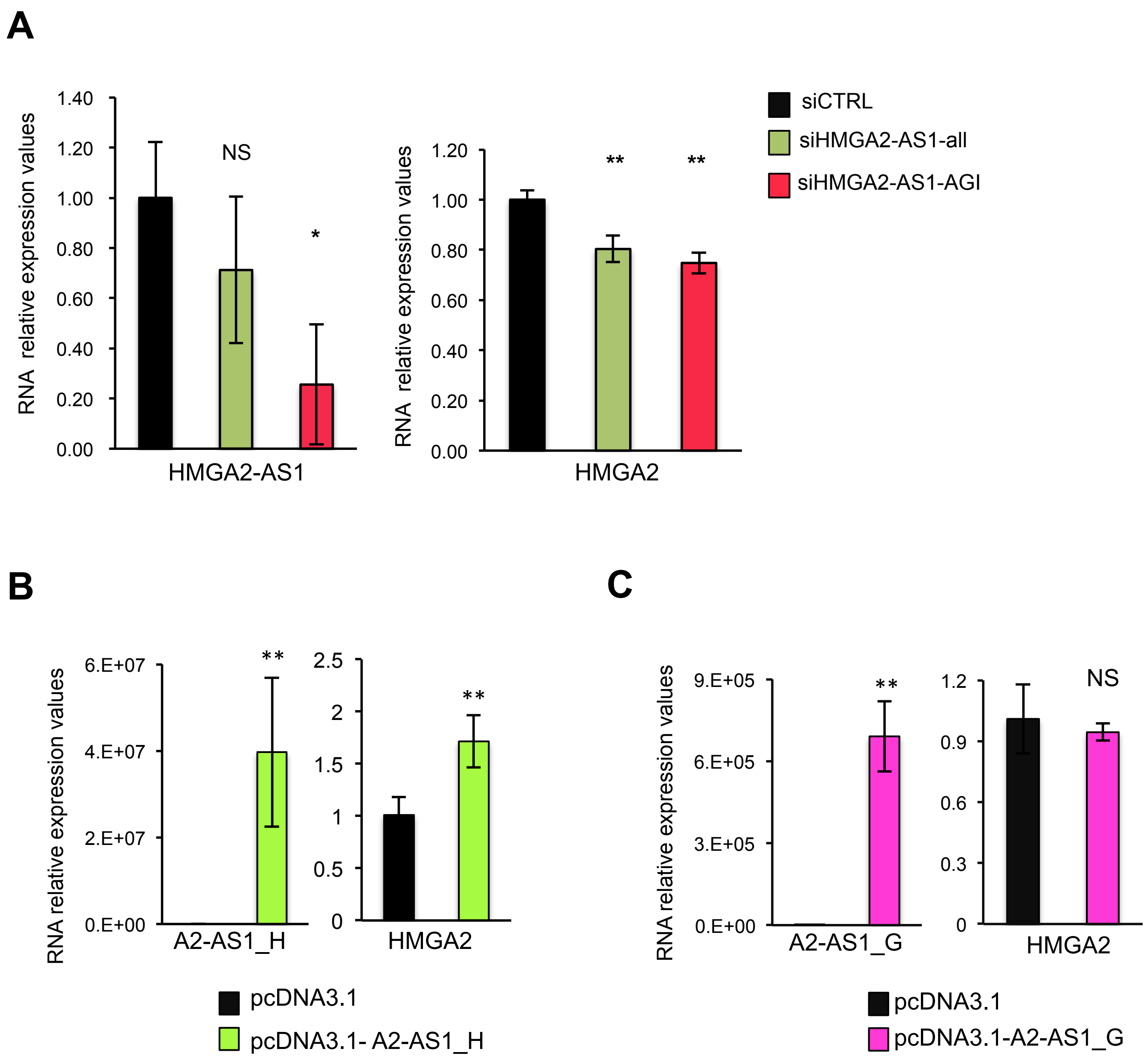

Supplemental Figure 4. HMGA2-AS1 regulates the expression of HMGA2. (A) qRT-PCR analysis of HMGA2-AS1 and HMGA2 after 72 hours of siHMGA2-AS1-all and siHMGA2-AS1-AGI silencing in PC3 cell line. Primers used to detect HMGA2-AS1 amplify together A2-AS1_A, A2-AS1_G and A2-AS1_I. GAPDH was used for normalization. The data are compared to siCTRL and are presented as the mean±SD (n=3), *p≤0.05, ***p*≤0.01; **(B and C)** Levels of A2-AS1_H and HMGA2 (B), and levels of A2-AS1_G and HMGA2 (C), were detected via qRT-PCR in PANC1 cells, transfected with pcDNA3.1 (control) and pcDNA3.1-A2-AS1_H (B) or pcDNA3.1-A2-AS1_G (C) for 30 hours. GAPDH was used for normalization, the data are compared to pcDNA3.1 and are presented as the mean±SD (*n*=3), NS: Not Significant, ***p*≤0.01; two-tailed Student's *t*-test.

Supplemental Figure 5

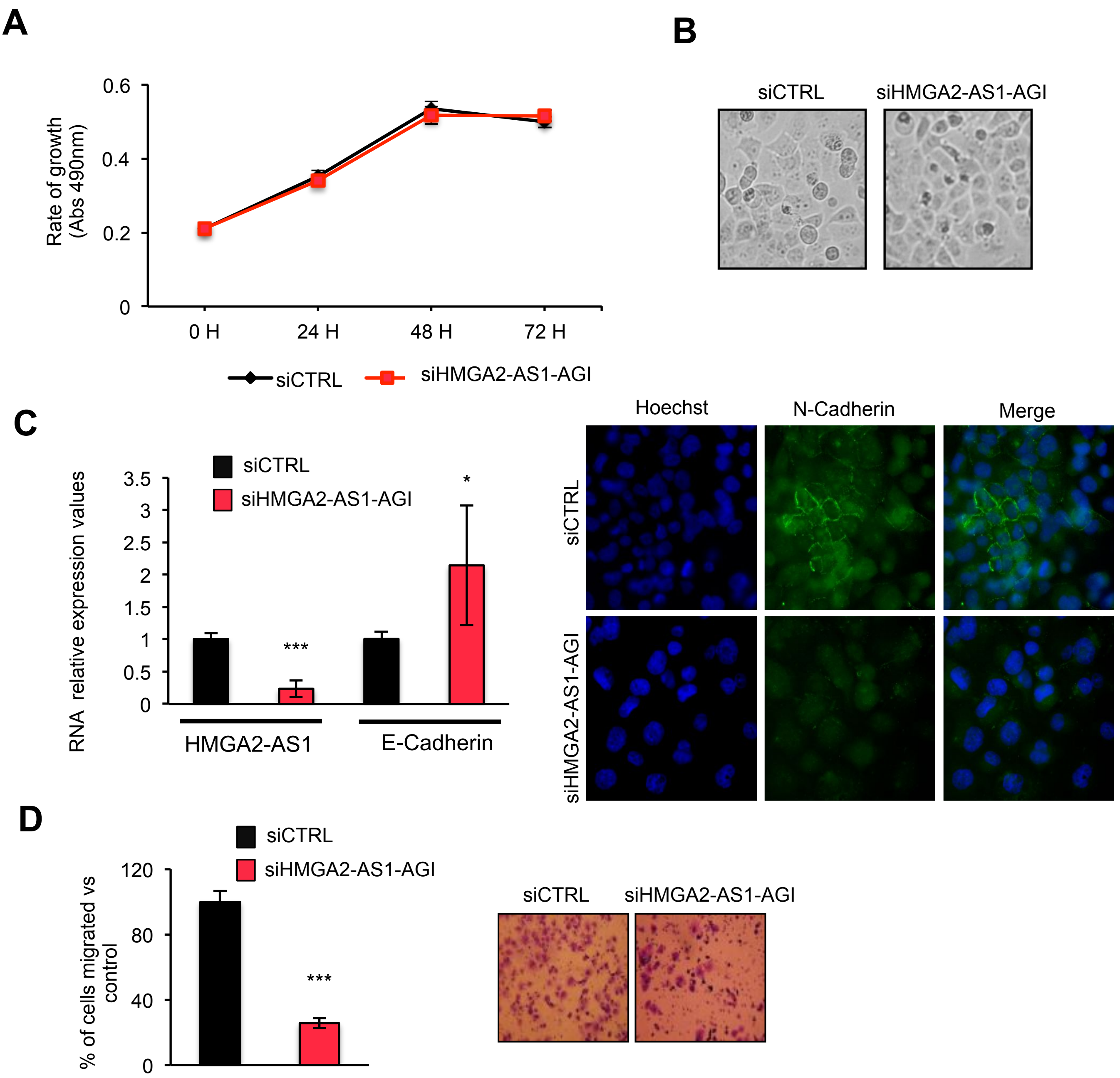

Supplemental Figure 5. Natural antisense lncRNAs from *HMGA2-AS1 locus* are involved in cancer promotion. (A) MTS proliferation assay in PANC1 cells silenced or not for HMGA2-AS1 transcript variants with siHMGA2-AS1-AGI. The data are presented as mean±SD (*n*=4). **(B)** Representative pictures of cell morphology of PANC1 cell culture in control condition and after 72 hours of siHMGA2-AS1-AGI transfection. **(C)** qRT-PCR analysis of HMGA2-AS1 silencing and the epithelial marker E-cadherin after 72 hours of siHMGA2-AS1-AGI silencing in PANC1 cell line. Primers used to detect HMGA2-AS1 amplify together A2-AS1_A, A2-AS1_G and A2-AS1_I. GAPDH was used for normalization. The data are compared to siCTRL and are presented as the mean±SD (n=3), *p≤0.05, ***p≤0.001; two-tailed Student's t-test. On the right, representative immunofluorescence images of the mesenchymal marker N-cadherin localization in PANC1 control and silenced cells with siHMGA2-AS1-AGI. Images were taken at 40X magnification. **(D)** Transwell assay of PANC1 cells silenced with siHMGA2-AS1-AGI for 72 hours. On the left, quantification of the transwell assay. The data are represented as the mean of percentage of migrated cells respect to siCTRL ±SD (n=4), ***p≤0.001; two-tailed Student's t-test. On the right, representative images of cells migrated across the porous membrane stained with crystal violet.

Supplemental Figure 6

A

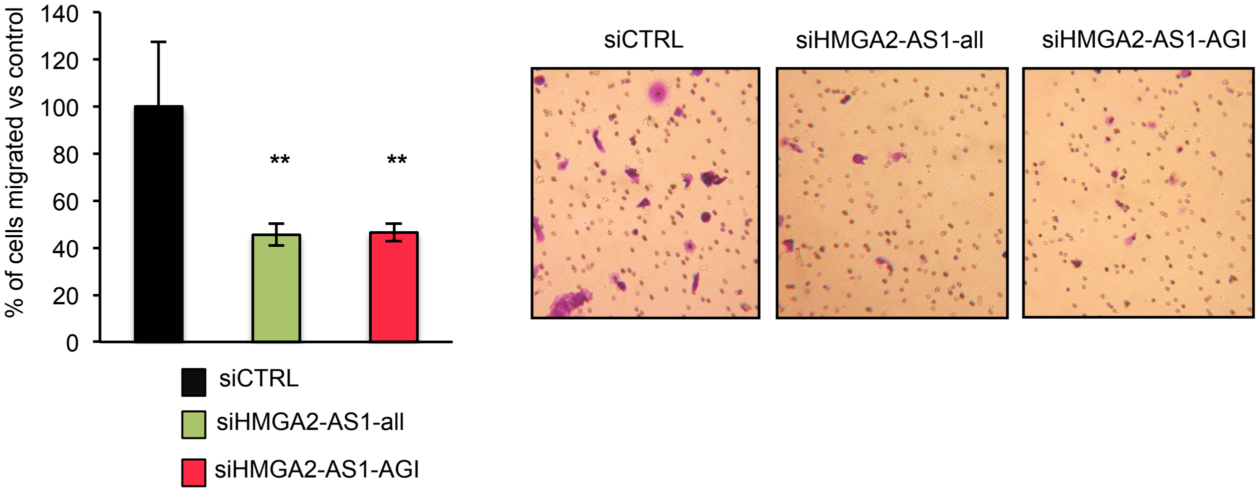

B

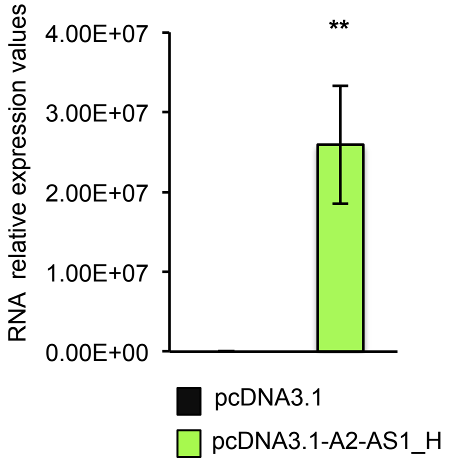

C

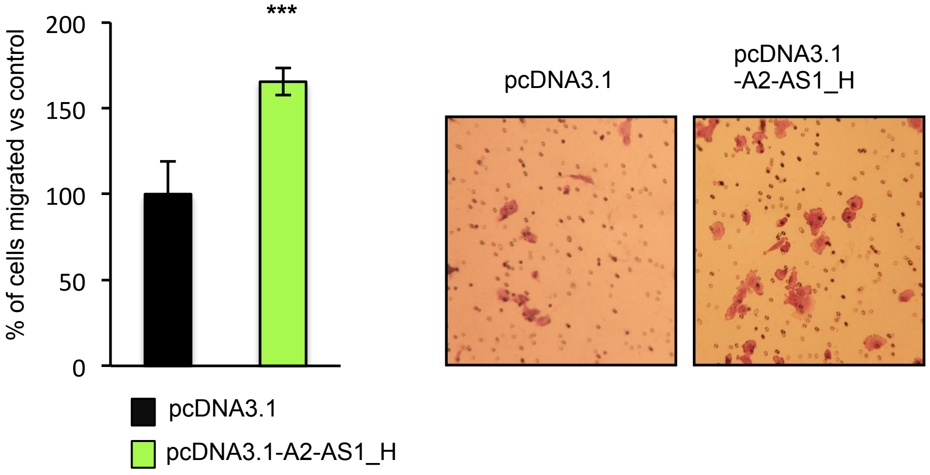

Supplemental Figure 6. Natural antisense lncRNAs from *HMGA2-AS1 locus* modulates cancer cell migration. (A) Transwell assay of PC3 cells silenced with siHMGA2-AS1-all and siHMGA2-AS1-AGI for 72 hours. On the left, quantification of the transwell assay. The data are represented as the mean of percentage of migrated cells respect to siCTRL ±SD (n=4), $^{**}p \leq 0.01$; two-tailed Student's t-test. On the right, representative images of cells migrated across the porous membrane stained with crystal violet. **(B)** qRT-PCR analysis of A2-AS1_H transcript variants in BX-PC3 cells transfected with pcDNA3.1 (control) and pcDNA3.1-A2-AS1_H for 30 hours. GAPDH was used for normalization. The data are compared to siCTRL and are presented as the mean±SD (n=3), $^{**}p \leq 0.01$; **(C)** Transwell assay of BX-PC3 cells treated as in (B). On the left, quantification of the transwell assay. The data are represented as the mean of percentage of migrated cells respect to siCTRL ±SD (n=4), ***p≤0.001; two-tailed Student's t-test. On the right, representative images of cells migrated across the porous membrane stained with crystal violet.
