# Supplementary figures and images for: HMGA2 Antisense Long Non-coding RNAs as New Players in the Regulation of HMGA2 Expression and Pancreatic Cancer Promotion
Source: Front Oncol. 2020 Jan 17;9:1526. doi: 10.3389/fonc.2019.01526 (PMC6978849; doi:10.3389/fonc.2019.01526)

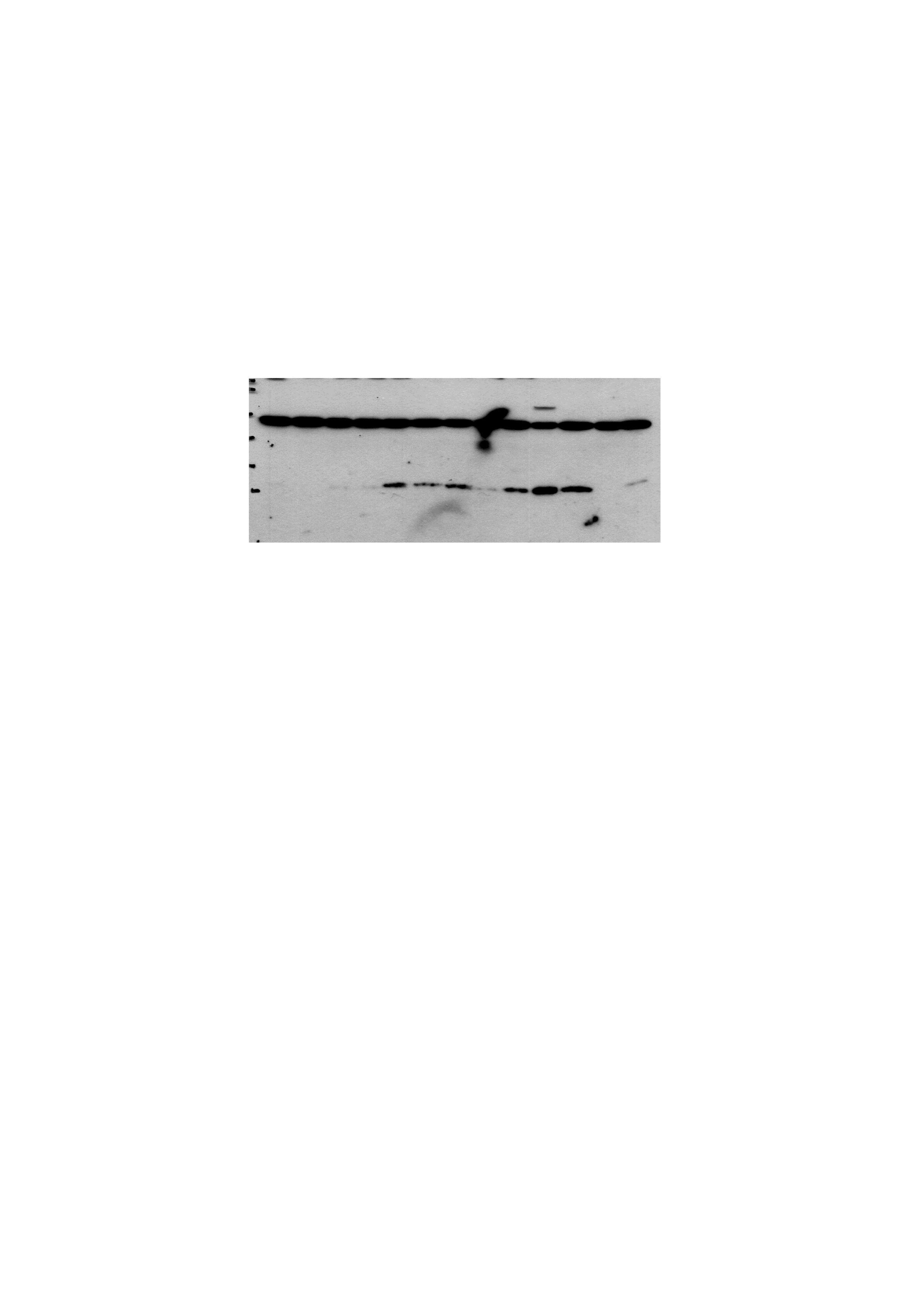

Supplement: Supplementary file 2 [file Image_1.JPEG]

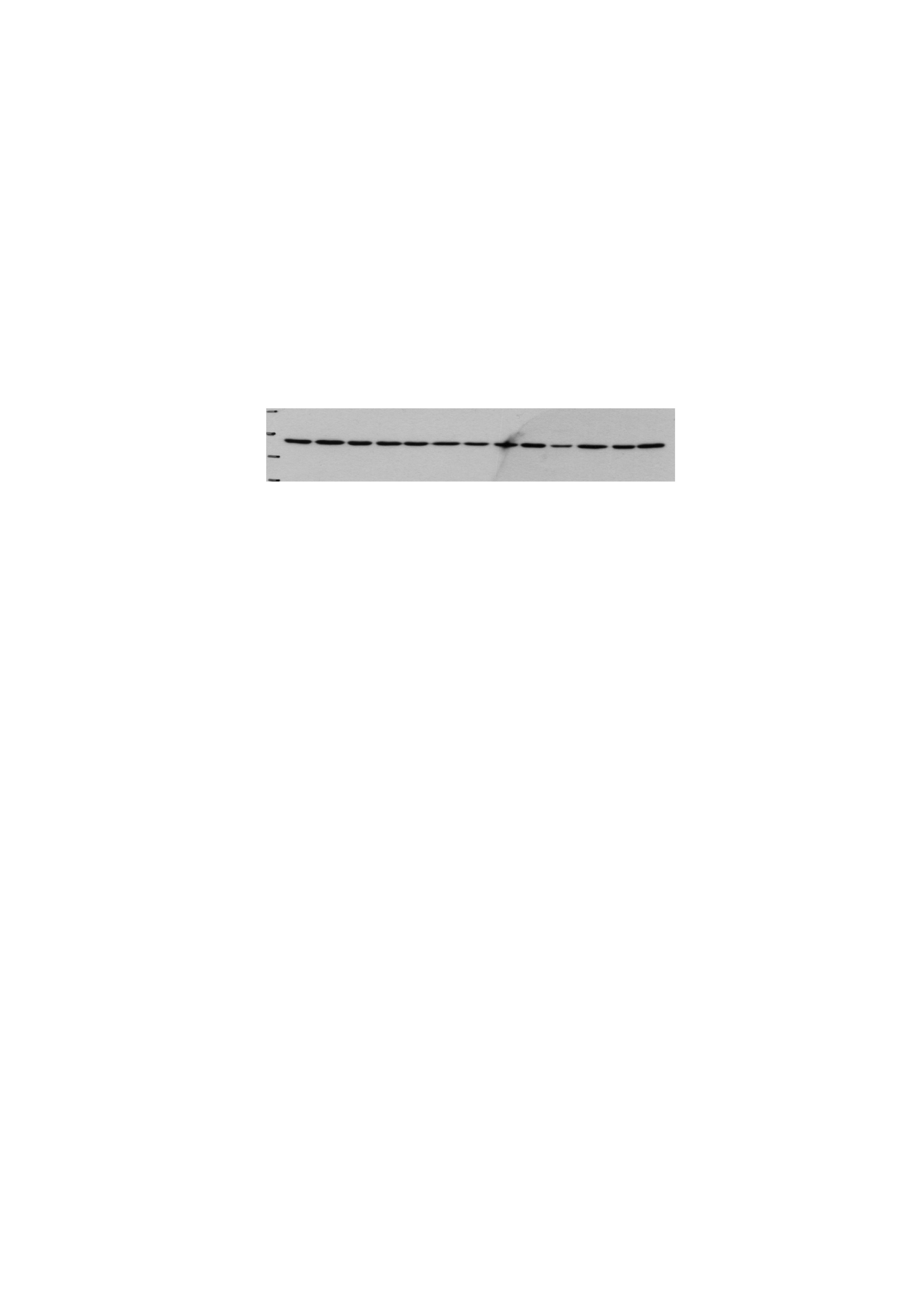

Supplement: Supplementary file 3 [file Image_2.JPEG]

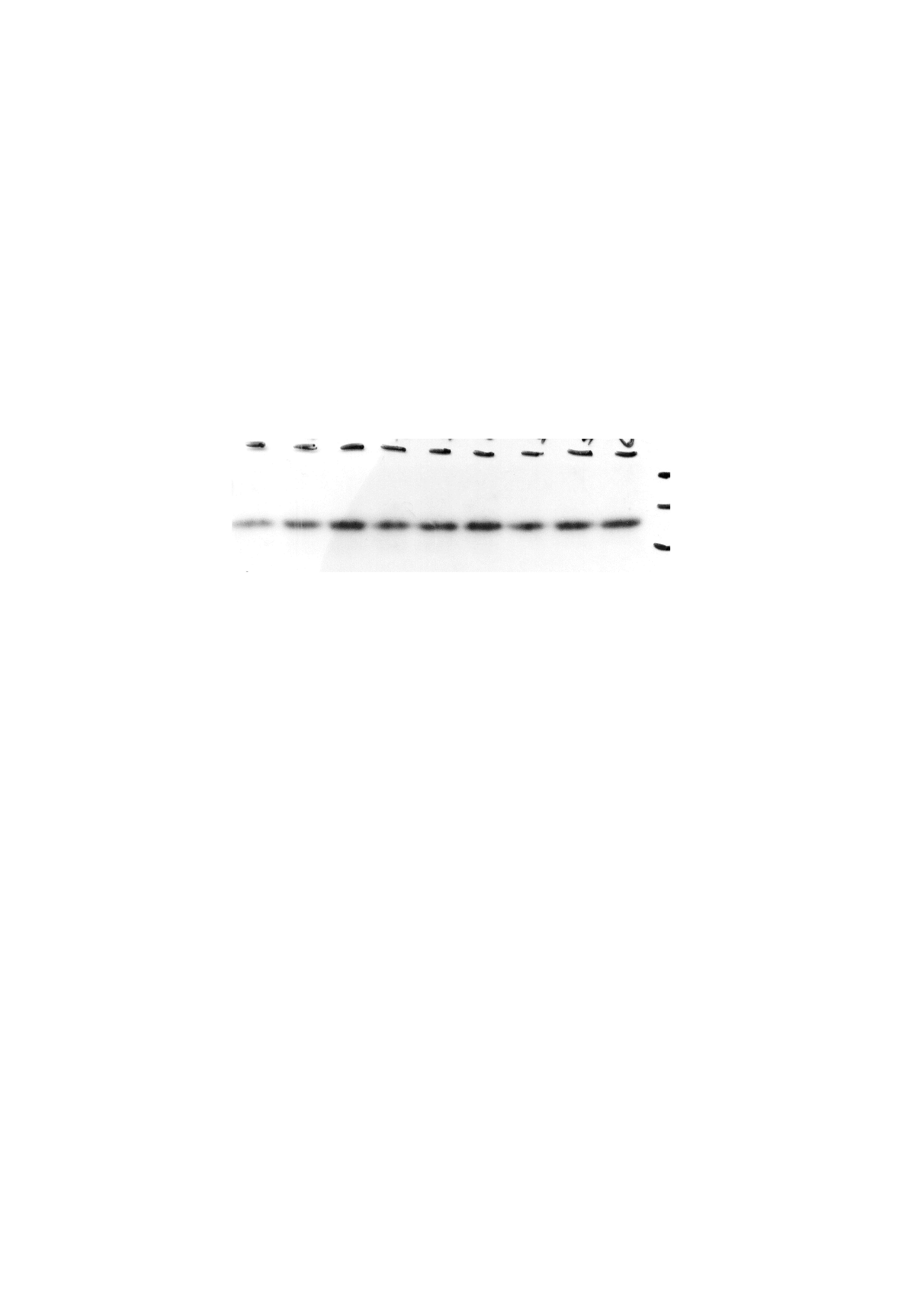

Supplement: Supplementary file 4 [file Image_3.JPEG]

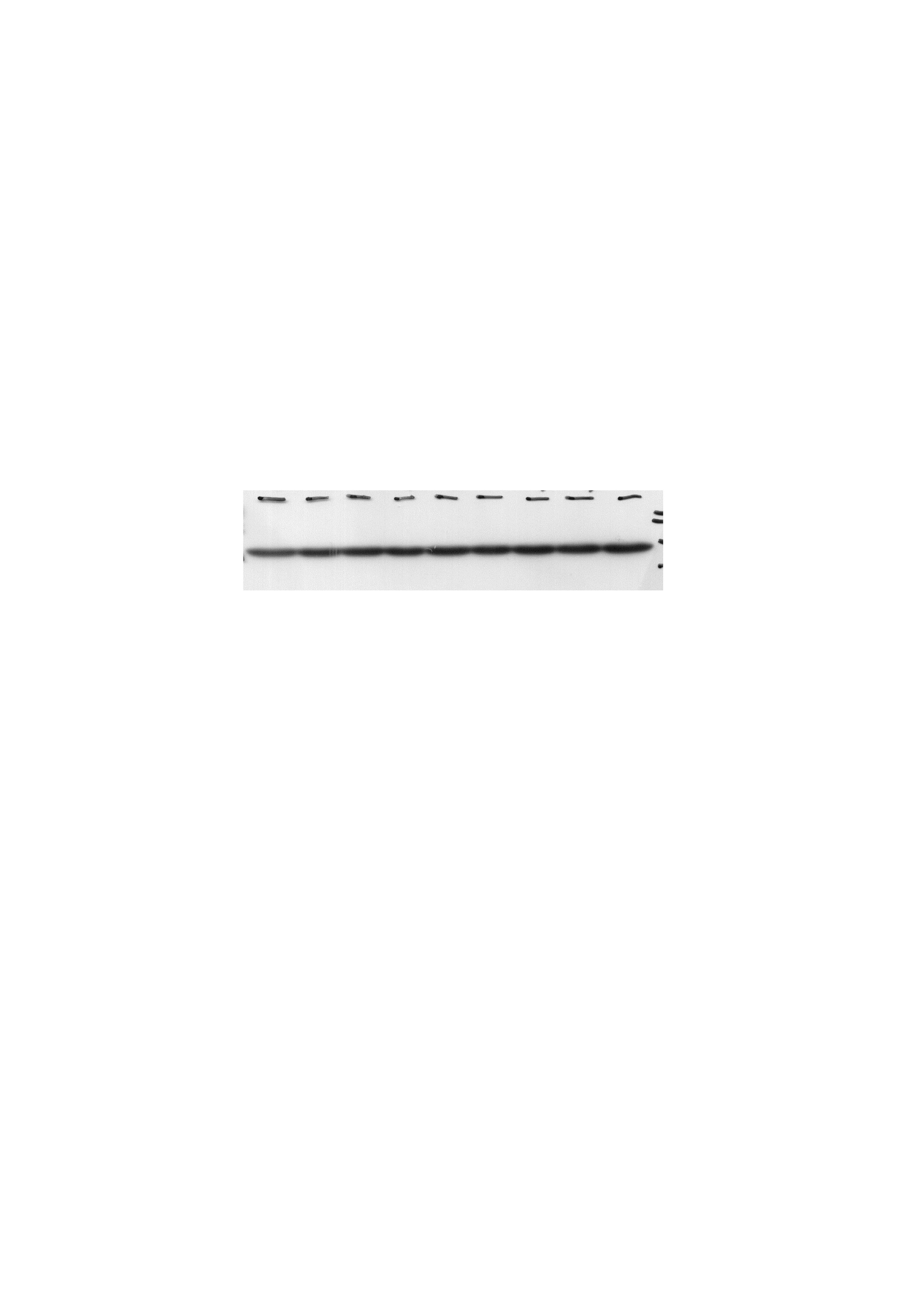

Supplement: Supplementary file 5 [file Image_4.JPEG]

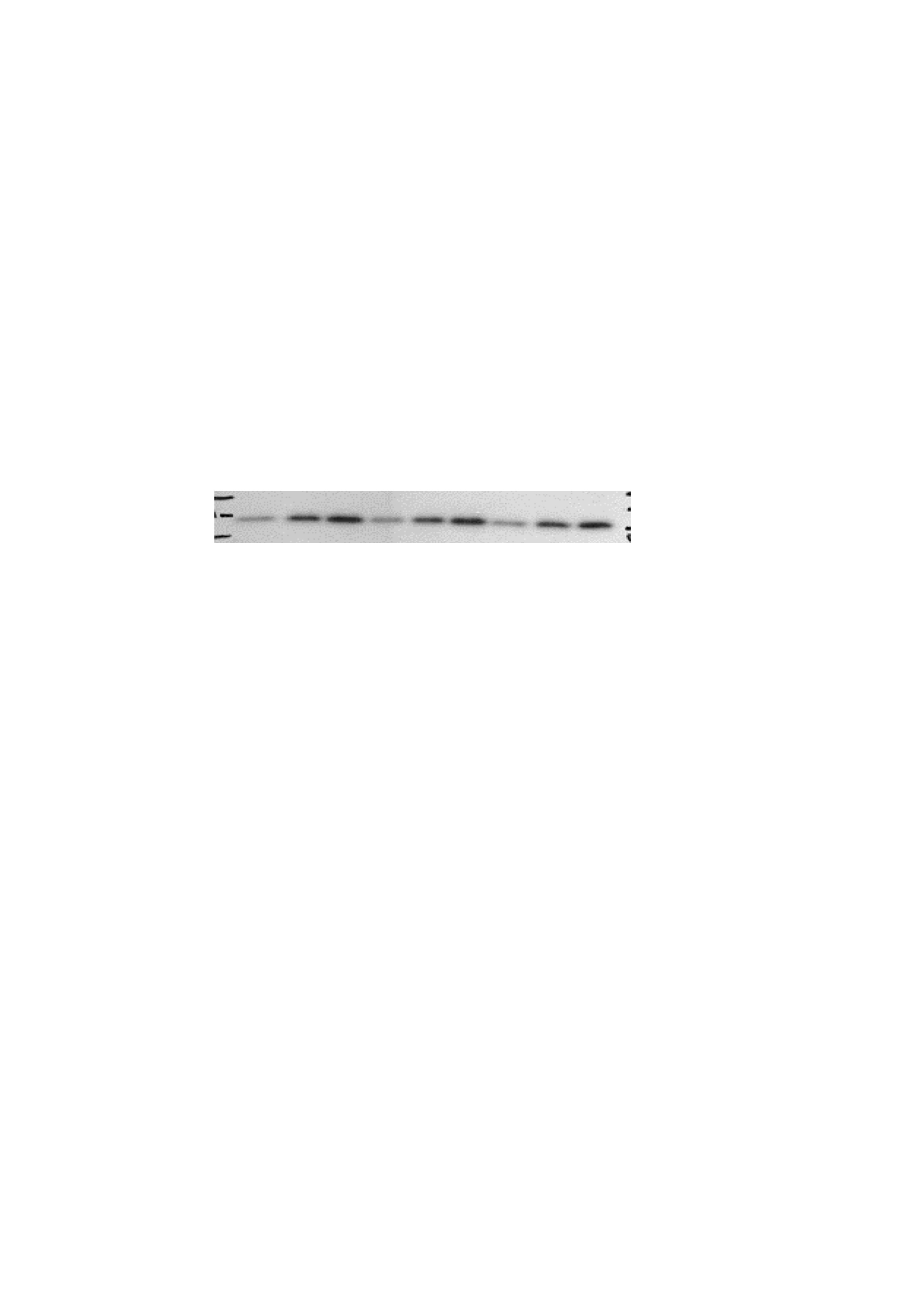

Supplement: Supplementary file 6 [file Image_5.JPEG]

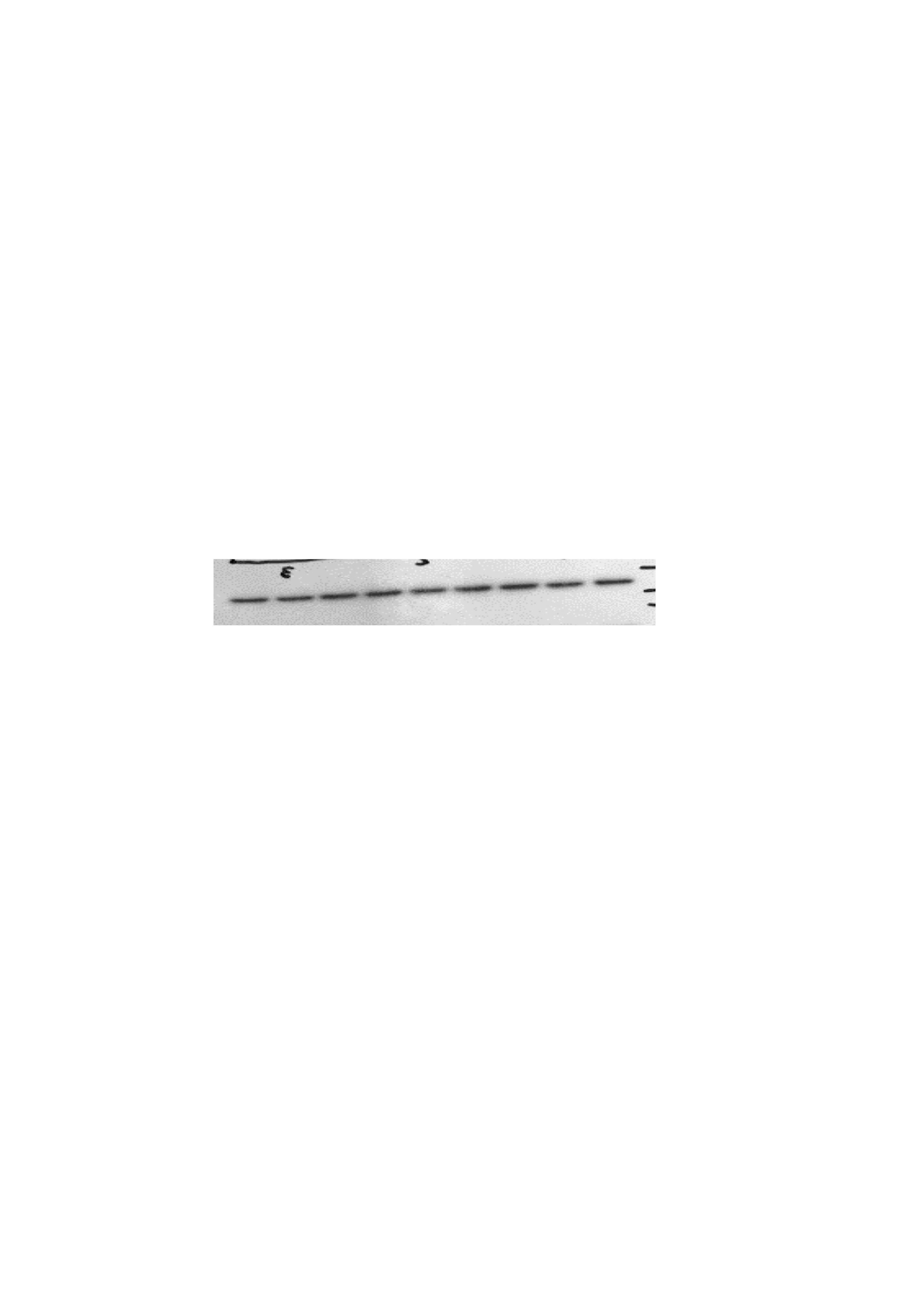

Supplement: Supplementary file 7 [file Image_6.JPEG]

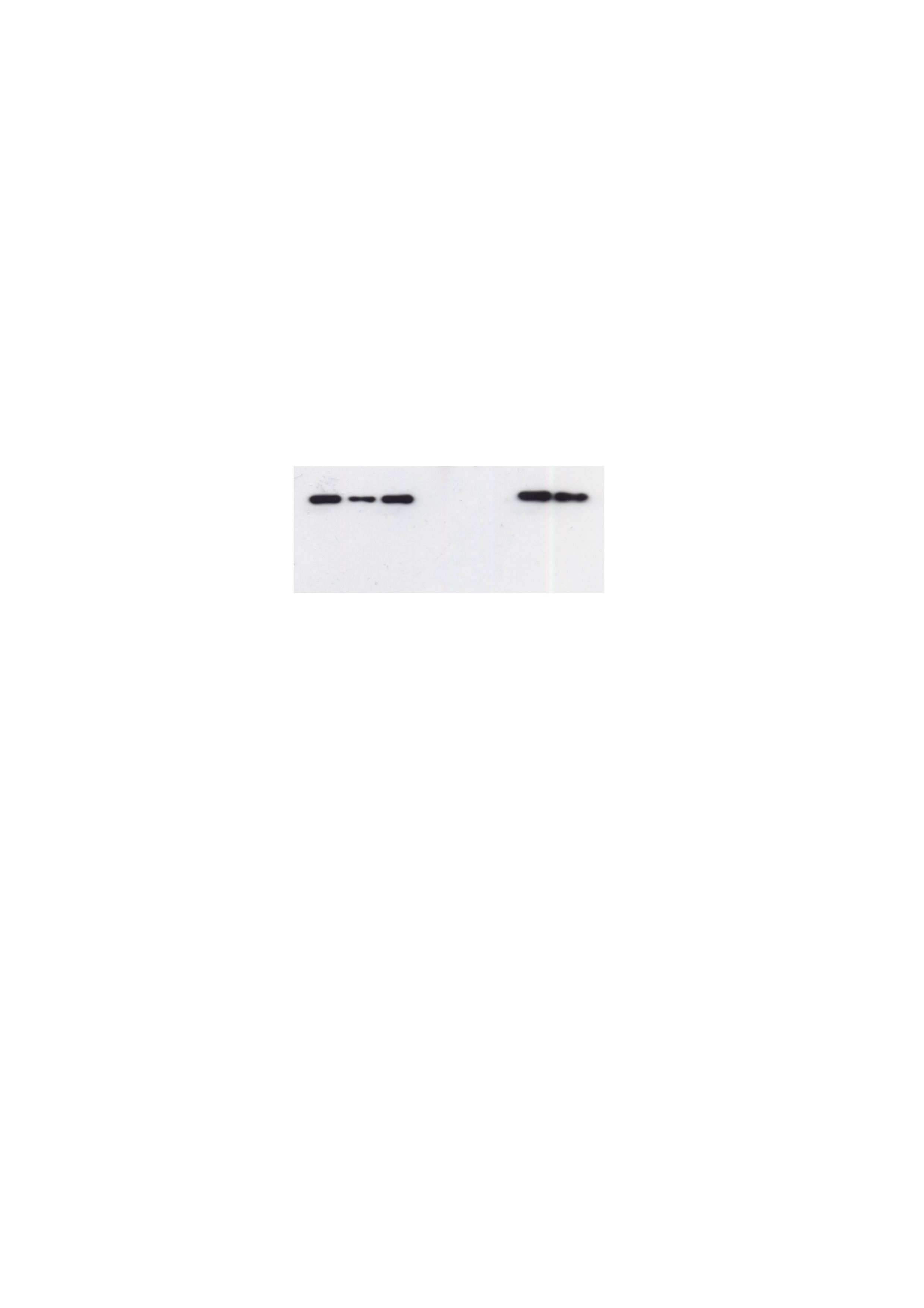

Supplement: Supplementary file 8 [file Image_7.JPEG]

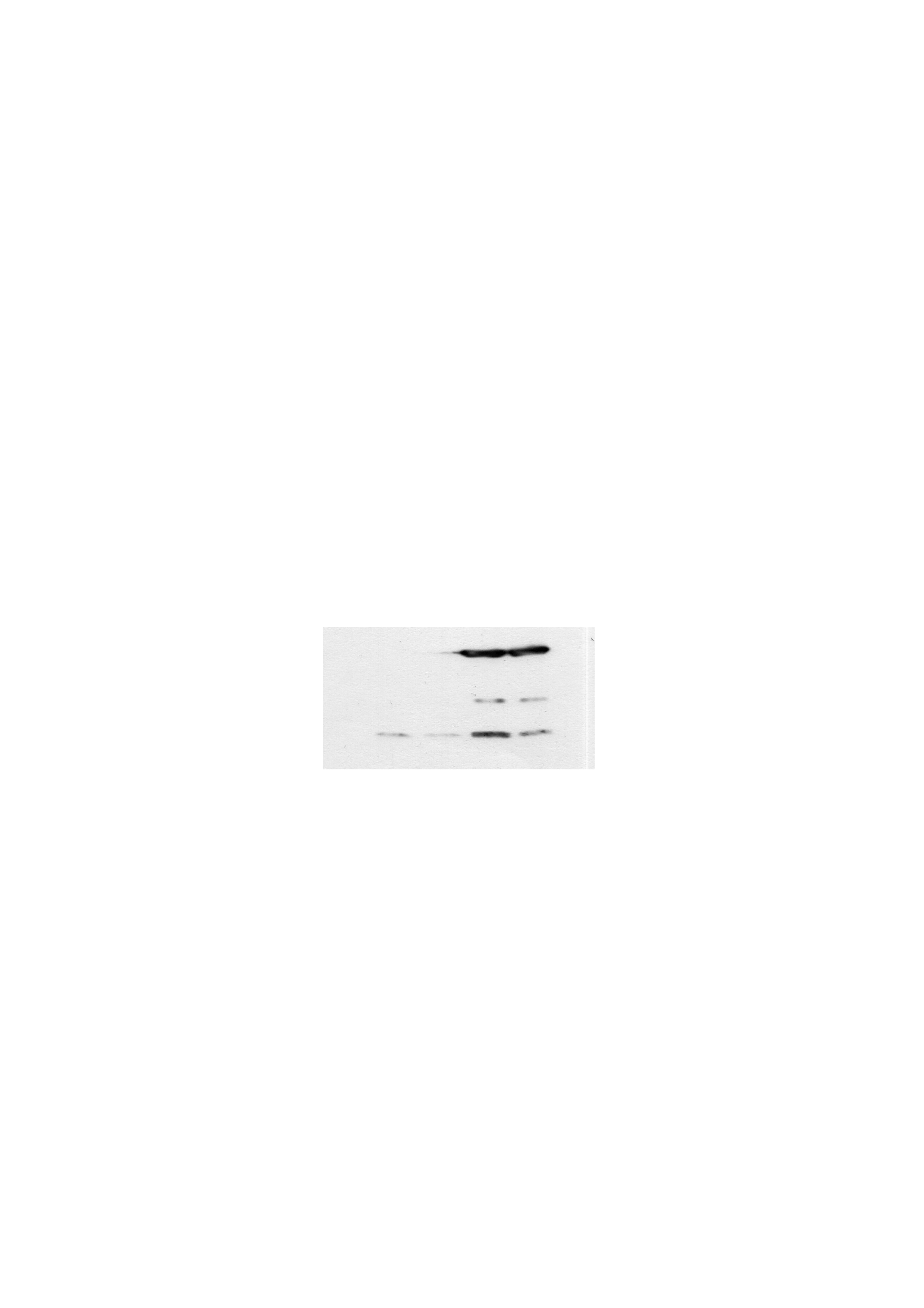

Supplement: Supplementary file 9 [file Image_8.JPEG]

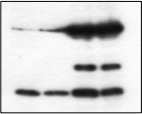

Supplement: Supplemental Images 1–9 — Full scans of western blots. [file Image_9.JPEG]
